# Temoporfin-Conjugated PEGylated Poly(*N*,*N*-dimethylacrylamide)-Coated Upconversion Colloid for NIR-Induced Photodynamic Therapy of Pancreatic Cancer

**DOI:** 10.1021/acs.biomac.4c00317

**Published:** 2024-06-18

**Authors:** Oleksandr Shapoval, Vitalii Patsula, David Větvička, Hana Engstová, Viktoriia Oleksa, Martina Kabešová, Taras Vasylyshyn, Pavla Poučková, Daniel Horák

**Affiliations:** † Institute of Macromolecular Chemistry, Czech Academy of Sciences, Heyrovského Nám. 2, 162 00 Prague 6, Czech Republic; ‡ First Faculty of Medicine, Charles University, Salmovská 1, 120 00 Prague 2, Czech Republic; § Institute of Physiology, Czech Academy of Sciences, Vídeňská 1083, 142 20 Prague 4, Czech Republic

## Abstract

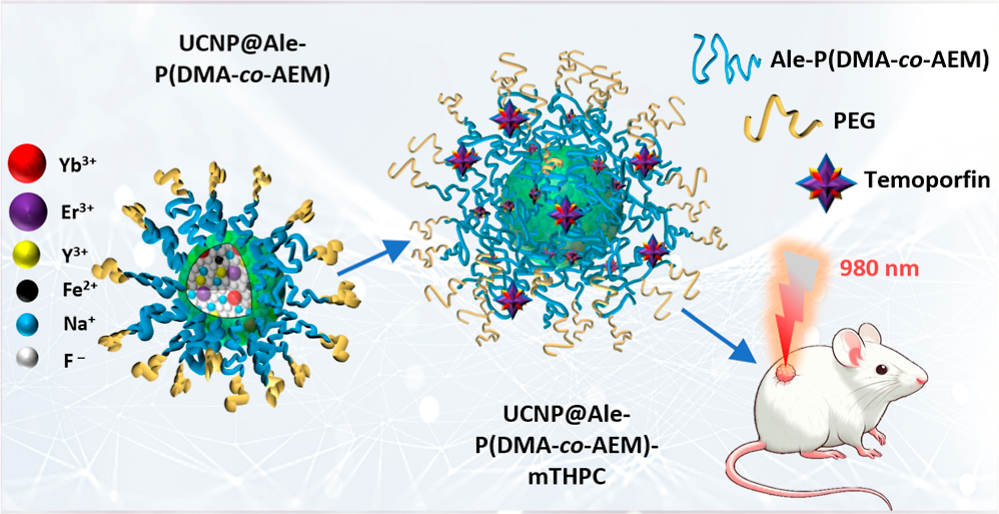

Photodynamic therapy (PDT) has the potential to cure pancreatic cancer with minimal side effects. Visible wavelengths are primarily used to activate hydrophobic photosensitizers, but in clinical practice, these wavelengths do not sufficiently penetrate deeper localized tumor cells. In this work, NaYF_4_:Yb^3+^,Er^3+^,Fe^2+^ upconversion nanoparticles (UCNPs) were coated with polymer and labeled with *meta*-tetra(hydroxyphenyl)chlorin (mTHPC; temoporfin) to enable near-infrared light (NIR)-triggered PDT of pancreatic cancer. The coating consisted of alendronate-terminated poly[*N*,*N*-dimethylacrylamide-*co*-2-aminoethylacrylamide]-*graft*-poly(ethylene glycol) [P(DMA-AEM)-PEG-Ale] to ensure the chemical and colloidal stability of the particles in aqueous physiological fluids, thereby also improving the therapeutic efficacy. The designed particles were well tolerated by the human pancreatic adenocarcinoma cell lines CAPAN-2, PANC-1, and PA-TU-8902. After intratumoral injection of mTHPC-conjugated polymer-coated UCNPs and subsequent exposure to 980 nm NIR light, excellent PDT efficacy was achieved in tumor-bearing mice.

## Introduction

Pancreatic cancer is one of the world’s most common cancers and causes of death.^1^ The number of new cases, which have a very poor prognosis, is increasing by approximately 1% a year, yet only 24% of people survive 1 year and 9% live for 5 years.^2^ Conventional treatments for pancreatic cancer include surgical resection and chemo- and radiotherapy.^3^ While surgery remains the first choice of treatment for this disease, resection is considered to be effective for less than 20% of patients at initial diagnosis and is not an option for more than 80% of patients with locally advanced or metastatic disease.^4^ In patients with inoperable pancreatic cancer, aggressive chemotherapy with the pyrimidine nucleoside analogues capecitabine and 5-fluorouracil is the primary treatment, but its clinical efficacy is still unsatisfactory.^5^ As a result, the median overall survival of patients with metastatic disease is less than one year. The limitations of current strategies for treating pancreatic cancer highlight the need to explore new research directions for future therapies.^6^

Photodynamic therapy (PDT) is receiving increasing attention as an alternative minimally invasive therapy for inoperable patients due to its efficacy against chemo- and radioresistant cells.^7,8^ In addition, PDT has already been approved by the Food and Drug Administration for the oncological treatment of pancreatic, lung, skin, head, neck, and prostate cancers.^9^ Treatment consists of the administration and accumulation of a photosensitizing agent in tumors, which, when activated by light, causes the death of tumor cells by generating reactive oxygen species (ROS).^10^ The advantage of PDT is that it does not cause cumulative toxicity associated with radiotherapy because the light used is nonionizing. For PDT to be well applicable to deep tumors, photosensitizers should have a high extinction coefficient in the near-infrared (NIR) spectral region.^11^ NIR light then overcomes the limited penetration of UV/vis light deep into tissues, where therapeutic potential can be fully exploited through noninvasive deep tissue imaging and drug delivery.^12,13^ In particular, benzoporphyrins have proven to be effective and safe agents for the treatment of pancreatic cancer.^14,15^

Another promising and powerful photosensitizer for PDT is the porphyrin derivative *meta*-tetra(hydroxyphenyl)chlorin (mTHPC; temoporfin; Foscan). It has enhanced light absorption in the red region (∼650 nm), minimal dark toxicity, increased phototoxicity, and is chemically pure.^16^ mTHPC has been approved by the European Medical Agency for the palliative treatment of head and neck cancer and has also been investigated as a primary treatment for skin, prostate, thoracic, brain, biliary, breast, and pancreatic cancers.^17,18^ The first clinical study of PDT in pancreatic cancer using mTHPC photoactivated at higher wavelengths (∼650 nm) documented a deep tumoricidal effect (10 mm) associated with tumor necrosis and concomitant reduction in morbidity and mortality.^19,20^ However, PDT still suffers from several limitations associated with photosensitizers, such as the lack of an optimal biological spectral window for tissue penetration and poor solubility, leading to fluorescence quenching, low target delivery, and low ROS generation efficiency.

Chemical modifications of mTHPC and its conjugation to polymers or inorganic particles can overcome the above obstacles. Another advantage of this conjugation method is high photosensitizer loading, targeting to the desired site, increased solubility and biocompatibility, and enhanced photochemical properties.^21−24^ The conjugation of mTHPC with polymers,^25^ glucose,^26^ folic acid,^27^ and ibuprofen^28^ increased drug selectivity against tumors and overcame post-treatment side effects. Nanocarriers based on polymers, lipids, or inorganic nanoparticles facilitated the administration of mTHPC and enabled the tuning of its pharmacokinetics.^18^

Upconverting nanoparticles (UCNPs) have great potential in PDT due to their low autofluorescence, color purity, high chemical and thermal photostability, ease of surface functionalization, and high signal-to-noise ratio.^29,30^ UCNPs enable the conversion of low-energy, high-penetrance NIR light to high-energy UV/vis light.^31^ mTHPC-conjugated UCNPs allowed effective treatment of glioblastoma.^32^ Modification and chemical conjugation of mTHPC with UCNPs killed up to 70% of the cancer cells after irradiation at 980 nm.^33^ However, PDT studies of mTHPC-conjugated UCNPs in tumor-bearing animal models are rather sparse, making it difficult to assess their benefit over temoporfin alone.

**Scheme 1:**
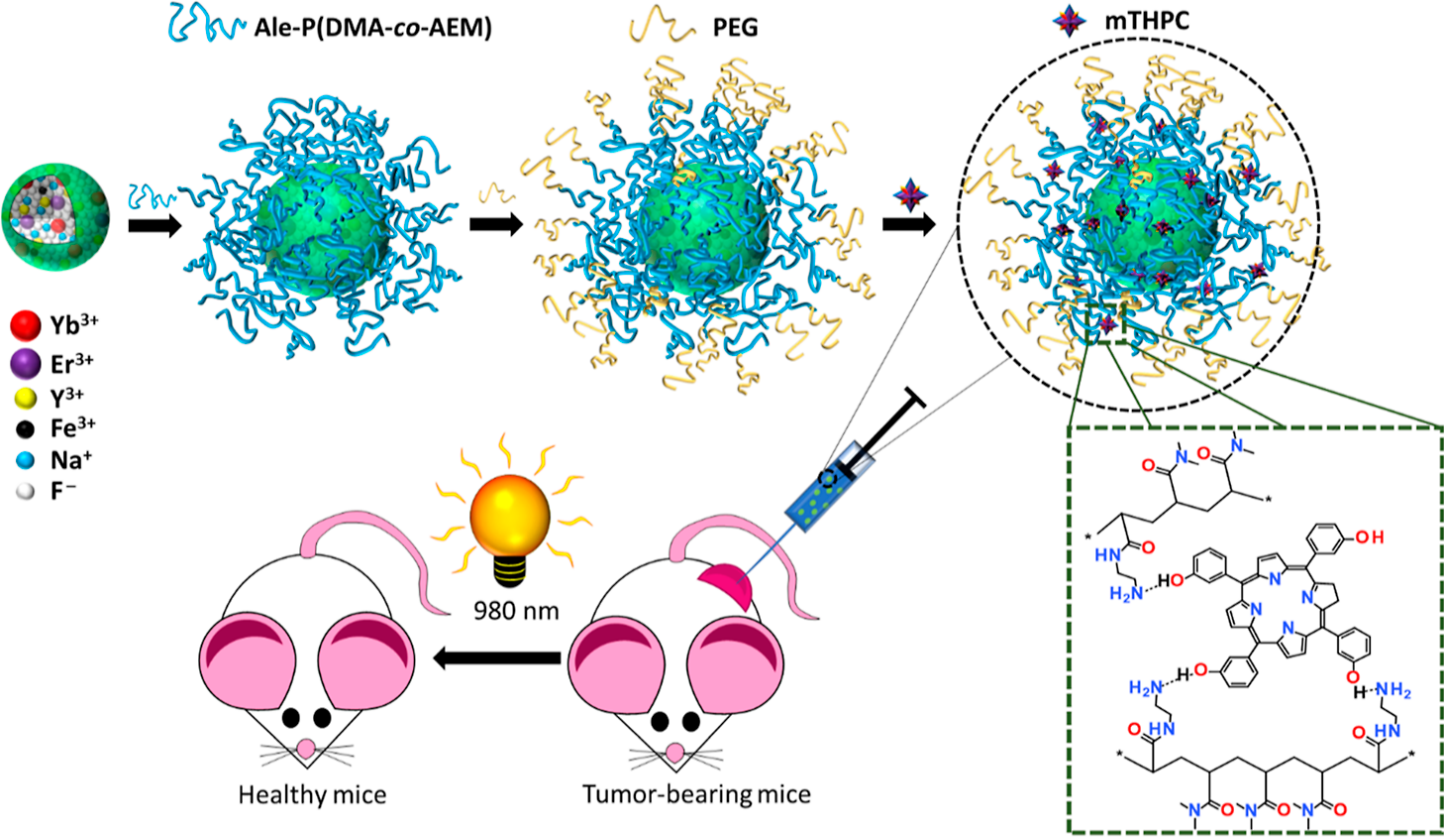
Schematic Representation of the Synthetic Procedures Used for the Preparation of the UCNP@Ale-P(DMA-AEM)-PEG-mTHPC Colloid and 980 nm NIR-Induced PDT of Pancreatic Adenocarcinoma in an Animal Model

Our recently published *in vivo* study of NIR-induced PDT of pancreatic adenocarcinoma growing subcutaneously in athymic mice using the photosensitizer mTHPC conjugated to poly(methyl vinyl ether-*alt*-maleic acid)-coated UCNPs showed that the animals were not completely cured.^34^ Therefore, in this report, we focused on the design and synthesis of UCNPs coated with new mTHPC-conjugated alendronate-terminated poly[*N*,*N*-dimethylacrylamide-*co*-2-aminoethylacrylamide]-*graft*-poly(ethylene glycol) [P(DMA-AEM)-PEG-Ale] as an alternative to currently used photosensitizers for PDT of pancreatic cancer (Scheme 1). The designed coating not only improved the colloidal stability of the particles in physiological fluids but also enabled superior NIR-induced PDT under mild NIR irradiation.

## Experimental Section

### Materials

**Scheme 2:**
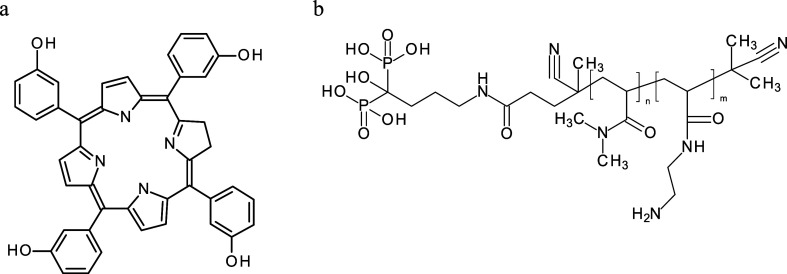
(a) *meta*-Tetra(hydroxyphenyl)chlorin and (b) Poly(*N*,*N*-dimethylacrylamide-*co*-2-aminoethylacrylamide)-alendronate

Octadec-1-ene (90%), chloride from yttrium (YCl_3_; 99%), erbium (ErCl_3_·6H_2_O; 99%), ytterbium (YbCl_3_; 99%), iron (FeCl_2_·4H_2_O), ammonium fluoride (99.99%), *N*,*N*-diisopropylethylamine (DIPEA; ≥99%), 2-(dodecylthiocarbonothioylthio)-2-methylpropionic acid [chain transfer agent (CTA); 98%], 4-(dimethylamino)pyridine (99%), *N*,*N*′-dicyclohexylcarbodiimide (DCC; 99%), *N*-hydroxysuccinimide (NHS; 98%), *N*,*N*-dimethylacrylamide (DMA; 99%), 2,2′-azobis(2-isobutyronitrile) (AIBN), 4,4′-azobis(4-cyanovaleric acid) (ACVA), xylenol orange, 30% hydrogen peroxide, citric acid, Triton X-100, 1,3-diphenylisobenzofuran (DPBF), methylene blue, Dulbecco’s modified Eagle medium (DMEM), and phosphate-buffered saline (PBS; pH 7.4) were purchased from Sigma-Aldrich (St. Louis, MO, USA). Ethanol (99.8%), hexane (99.9%), methanol (99.9%), hydrochloric acid (35%), ethyl acetate (99.9%), sodium hydroxide, sodium chloride, and oleic acid were purchased from Lach-Ner (Neratovice, Czech Republic). All of the other chemicals were obtained from commercial sources and used without further purification. *meta*-Tetra(hydroxyphenyl)chlorin (mTHPC; temoporfin; Scheme 2a) was obtained from MedChemExpress (Monmouth Junction, NJ, USA). *N*,*N*-Dimethylformamide (DMF; 99.8%) was obtained from Iris Biotech (Marktredwitz, Germany). *N*-Succinimidyl-activated monomethyl poly(ethylene glycol) (PEG-NHS; *M*_n_ = 5000 g/mol) was purchased from Rapp Polymere (Tuebingen, Germany). *tert*-Butyl[2-(acryloylamino)ethyl]carbamate (AEC-Boc) was prepared according to the literature^35^ and purified by column chromatography on silica gel using ethyl acetate/hexane (4:1 v/v) as the eluent. Artificial lysosomal fluid (ALF; pH 4.5) was prepared as described in the literature.^36^ Artificial extracellular tumor fluid (AETF) was prepared from artificial cerebrospinal fluid by adjusting the pH to 6.5 and the H_2_O_2_ level to 100 μM using 0.1 M aqueous citric acid and 30 wt % H_2_O_2_ solution.^37^ The sodium salt trihydrate of (4-amino-1-hydroxy-1-phosphonobutyl)phosphonic acid (alendronate; Ale) was obtained from TCI (Tokyo, Japan). Distilled demineralized water (conductivity <0.1 μS/cm) obtained by reverse osmosis with UV treatment (Milli-Q Gradient A10 system; Millipore; Molsheim, France) was used throughout the experimental work.

### Synthesis of NaYF_4_:Yb^3+^,Er^3+^,Fe^2+^ Nanoparticles

Colloidal NaYF_4_:Yb^3+^,Er^3+^,Fe^2+^ nanoparticles (UCNPs) used in this report were prepared as follows. Briefly, 2 mmol of yttrium(III), ytterbium(III), erbium(III), and iron(II) chlorides (1.2:0.4:0.3:0.1 mol/mol/mol/mol, respectively) and oleic acid (24 mL) were dissolved in octadec-1-ene (30 mL) at 170 °C for 30 min under an Ar atmosphere.^34^ The solution was then cooled to room temperature (RT) to allow the addition of 16 mL of methanolic solution of NaOH (5 mmol) and NH_4_F (8 mmol). The temperature was slowly increased to 70 °C to evaporate the methanol and subsequently to 300 °C for 1.5 h while stirring under an Ar atmosphere. The resulting UCNPs were separated by centrifugation (3460 rcf) for 30 min, washed in a hexane/ethanol mixture (1:1 v/v) four times, washed in water (14 mL each) eight times, and redispersed in water. For physicochemical characterization, a portion of the dispersion was vacuum-dried at RT for 3 days.

### Preparation of Alendronate-Terminated Poly(*N*,*N*-dimethylacrylamide-*co*-2-aminoethylacrylamide) [P(DMA-AEM)-Ale]

Alendronate-terminated poly(*N*,*N*-dimethylacrylamide-*co*-2-aminoethylacrylamide) (Scheme 2b) was prepared by ACVA-initiated and CTA-controlled reversible addition–fragmentation (RAFT) polymerization. Briefly, ACVA and CTA ([ACVA]/[CTA] = 1:4.5 mol/mol) were added to a solution of DMA and AEC-Boc (9:1 mol/mol; [ACVA]/[monomer]_total_ = 1:369 mol/mol) in ethanol and polymerized at 70 °C for 30 min under an Ar atmosphere. The poly(*N*,*N*-dimethylacrylamide-*co*-*tert*-butyl[2-(acryloylamino)ethyl]carbamate) [P(DMA-AEC-Boc)] was isolated by precipitation in hexane, and the CTA-end groups were removed by refluxing the polymer with AIBN in methanol; P(DMA-AEC-Boc) had *M*_w_ = 11 kg/mol and a narrow distribution (*M*_w_/*M*_n_ = 1.2). The terminal carboxyl groups of the copolymer were reacted with Ale using DCC/NHS chemistry. Finally, the Boc protecting groups were removed by treatment of P(DMA-AEC-Boc)-Ale with 3 M methanolic HCl. The resulting P(DMA-AEM)-Ale was purified by gel filtration in methanol on a Sephadex LH-10 column (Sigma-Aldrich).

### Modification of UCNPs with P(DMA-AEM)-Ale and Grafting with Poly(ethylene glycol)

An aqueous dispersion (2.4 mL) of the UCNPs (40 mg) was added to an aqueous solution (3.2 mL) of P(DMA-AEM)-Ale (80 mg) under sonication (Hielscher UP200S ultrasonic homogenizer; Teltow, Germany; 20% power) for 1 min, and the mixture was stirred at 80 °C for 18 h. The UCNP@Ale-P(DMA-AEM) nanoparticles were separated by centrifugation (13,170 rcf) for 45 min, washed twice with water (4 mL), and redispersed in a water/ethanol mixture (0.75 mL; 1:1 v/v), after which DIPEA (20 μL) was added, followed by mixing for 20 min (900 rpm). The particles were separated by centrifugation (13,171 rcf) for 30 min and washed with water (2 mL). Then, PEG-NHS (10.7 mg; 2.14 μmol) was added to the dispersion of UCNP@Ale-P(DMA-AEM) particles (10 mg) in DMF (0.75 mL), and the mixture was stirred (900 rpm) at RT for 17 h. The resulting UCNP@Ale-P(DMA-AEM)-PEG particles were separated by centrifugation (13,170 rcf) for 45 min, washed with ethanol (2 mL), and redispersed.

### Conjugation of mTHPC to UCNP@Ale-P(DMA-AEM)-PEG Nanoparticles

mTHPC (0.53 mg; 0.78 μmol) was added to the dispersion of UCNP@Ale-P(DMA-AEM)-PEG particles (7 mg) in ethanol (1 mL), and the mixture was stirred (900 rpm) at RT for 24 h in the dark. The resulting UCNP@Ale-P(DMA-AEM)-PEG-mTHPC particles were separated by centrifugation (13,170 rcf) for 45 min, washed with ethanol (2 mL) and water (2 mL), redispersed in water to the desired concentration, and stored at 5 °C in the dark.

### Characterization of Nanoparticles

The nanoparticle morphology was analyzed by a Tecnai Spirit G2 transmission electron microscope (TEM; FEI; Brno, Czech Republic).^38^ The particle size and distribution were calculated by counting at least 300 particles from the TEM micrographs using the open-source image processing software ImageJ version 1.52p (National Institutes of Health; Bethesda, MD, USA). The number- (*D*_n_), weight-average particle diameter (*D*_w_), and dispersity (*D̵*) were calculated as follows
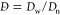
1
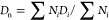
2
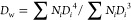
3where *D*_*i*_ and *N*_*i*_ are the diameter and number of the *i*-th particle, respectively.

The hydrodynamic particle diameter (*D*_h_), polydispersity (PD), and ζ-potential were measured via dynamic light scattering (DLS) and electrophoretic light scattering (ELS) on a ZSU 5700 Zetasizer Ultra apparatus (Malvern Instruments; Malvern, UK) at RT; *D*_h_ and PD were calculated from the intensity-weighted distribution function obtained via CONTIN analysis of the correlation function embedded in Malvern software. Thermogravimetric analysis (TGA) of the particles was performed in air with a PerkinElmer TGA 7 analyzer (Norwalk, CT, USA) over the temperature range of 30–700 °C at a constant heating rate of 5 °C/min. ^1^H and ^31^P NMR spectra were recorded with a Bruker AVANCE III 600 spectrometer (Bruker; Billerica, MA, USA). The content of temoporfin in the mTHPC-conjugated UCNPs was determined by an Evolution 220 UV–visible spectrophotometer (Thermo Fisher Scientific; Waltham, MA, USA) at 651 nm and compared to the calibration curve of mTHPC in ethanol.

Emission and excitation spectra were recorded in a Hellma 114F-QS cuvette (10 × 4 mm path length; Sigma-Aldrich) at RT on an FS5 Edinburgh Instrument spectrofluorometer (Edinburgh, UK) equipped with continuous (150 W) and pulsed xenon lamps and coupled with a CW 980 nm infrared diode laser as an excitation source with a nominal laser power of 2 W (MDL-III-980; beam size 5 × 8 mm^2^).

The generation of hydroxyl radicals by the Fenton reaction was analyzed spectrophotometrically using methylene blue. The particle dispersion (0.25 mL; 2 mg/mL) in 0.1 M PBS or water was mixed with 3.5 mM methylene blue solution in water (1.5 mL) at RT in the dark, which was followed by the injection of 30% unstabilized H_2_O_2_ (0.5 mL). The time dependence of methylene blue degradation corresponding to the production of hydroxyl radicals was monitored by a Specord 250 Plus UV–vis spectrophotometer (Analytik Jena; Jena, Germany) at 500–750 nm with careful stirring (250 rpm) for 2 min between measurements.

Singlet oxygen (^1^O_2_) generation was determined spectrophotometrically using a DPBF probe according to previous method.^34^ Briefly, an aqueous nanoparticle dispersion (0.1 mL; 2 mg/mL) was mixed with a freshly prepared 10 mM solution of DPBF in ethanol/water (50:50 v/v) in Hellma quartz cells (Sigma-Aldrich). Time-dependent irradiation was performed in the FS5 Edinburgh Instrument spectrofluorometer in the dark with a 980 nm laser (MDL-III-980-2W; 2.11 W/cm^2^) or a continuous 150 W xenon lamp at 650 nm. The DPBF absorbance was monitored with a Specord 250 Plus UV–vis spectrophotometer at 350–650 nm as a function of exposure time; samples were gently agitated (250 rpm) for 2 min between measurements; and the decrease in peak intensity at 415 nm was associated with ^1^O_2_ formation.

### Chemical Stability of the UCNPs and the UCNP@Ale-P(DMA-AEM)-PEG Colloid

A dispersion of particles (1 mg/mL) in the corresponding medium (0.01 M PBS, water, DMEM with 10% fetal bovine serum, ALF, or AETF) was added to a 2 mL plastic vial, which was sealed and incubated at 37 °C for 0–168 h while mixing (250 rpm). The zero point was determined after 5 min of aging. After that, the particles were separated by centrifugation (14,130 rcf) for 25 min, and the resulting supernatant was filtered (MWCO = 30 kg/mol) to remove residual particles. The content of dissolved fluorine was measured by a combined fluoride electrode (Thermo Fisher Scientific; Waltham, MA, USA) according to the manufacturer’s protocol. The leaching of free metal ions (the sum of rare-earth and iron ions) from the particles was determined by a Specord 250 Plus UV–vis spectrophotometer at 350–650 nm using xylenol orange as previously described.^39^ The supernatant (0.2 mL) was mixed with acetate buffered xylenol orange solution (2 mL; pH 5.8). The total metal ion concentration was directly proportional to the ratio of the absorbance at 570 and 450 nm determined from a calibration curve of 18 μM xylenol orange in acetate buffer (pH 5.8) containing different amounts of YCl_3_ (0–70 μM Y^3+^). The molar percentages of dissolved F^–^ (*X*_F_) and metal ions (*X*_Me_) were related to the total number of fluorine and metal ions in the UCNPs, respectively.

### *In Vitro* Cell Proliferation Assay

The human pancreatic adenocarcinoma cell lines Capan-2, PANC-1, and PA-TU-8902 were obtained from the German DSMZ collection of microorganisms and cell cultures (Leibniz Institute; Berlin, Germany). The cells were cultured at 37 °C in a 5% CO_2_ atmosphere in DMEM supplemented with glucose (4.5 g/L), 1 mM sodium pyruvate, 1% penicillin/streptomycin (Gibco; Waltham, MA, USA), and either 20% FBS for Capan-2 or 10% FBS for PANC-1 and PA-TU-8902 cells. Prior to the experiments, the cells were washed with PBS and incubated in 0.025% trypsin and 0.01% ethylenediaminetetraacetic acid in PBS for 5–10 min to facilitate detachment.

The cytotoxicity of the Ale-P(DMA-AEM)-PEG-coated UCNPs with or without conjugated mTHPC was evaluated using the XTT assay (Sigma-Aldrich) on the three above-mentioned pancreatic carcinoma cell lines according to the manufacturer’s protocol. In brief, harvested cells were resuspended in growth medium and seeded into 96-well plates (TPP Techno Plastic Products; Trasadingen, Switzerland; 1 × 10^4^ cells; 200 μL per well). The next day, the UCNP dispersion (50 μL; 0.001–0.3 mg/mL) was added, and the plates were placed in a CO_2_ incubator for 48 h. The supernatant (150 μL) was removed, and 25 μL of a mixture containing sodium 3′-[1-(phenylaminocarbonyl)-3,4-tetrazolium]-bis(4-methoxy-6-nitro)benzenesulfonic acid hydrate (XTT) and phenazine methosulfate was added to the plates, which was followed by a 2 h incubation. The absorbance of the samples was measured at 450 nm with a reference wavelength of 620 nm, using a Tecan Infinite F50 plate reader (Schoeller; Prague, Czech Republic).

### *In Vitro* Photodynamic Therapy

To assess photodynamic activity *in vitro*, PANC-1 cells were cultured at 37 °C in an atmosphere of 5% CO_2_ in DMEM supplemented with glucose (4.5 g/L), 1 mM sodium pyruvate, 1% penicillin/streptomycin, and 10% FBS. Cells (2 × 10^5^) were seeded on poly(l-lysine)-coated coverslips 2 days before the experiment. The particle dispersion (0.3 mg/mL) was incubated with cells for 12 h, excited at 980 nm wavelength with a Coherent 170 fs Chameleon pulsed laser at 40 mW for 30 min, and observed using a Leica SP 8 confocal microscope (Leica Microsystems; Wetzlar, Germany) in bright-field mode using a transmitted light detector.

To determine the viability of PANC-1 cells *in vitro* (2 × 10^5^ cells) after irradiation at 980 nm, they were cultured for 30 h as described above and incubated in the absence (control) and presence of UCNP@Ale-P(DMA-AEM)-PEG and UCNP@Ale-P(DMA-AEM)-PEG-mTHPC particles (0.3 mg per mL DMEM) at 37 °C for 12 h in a 5% CO_2_ atmosphere. The cells were irradiated in the dark with a MDL-III-980 laser (2 W; 0.7 W/cm^2^) for various durations, and their viability was determined by staining with 0.4% trypan blue (Thermo Fisher Scientific). The fraction of live cells was counted on a LUNA-II automated cell counter (Logos Biosystems; Anyang-si, South Korea).

### Hemolysis Assay

Red blood cells (RBCs) isolated from mice by retroorbital puncture were used to evaluate the hemolytic activity of the nanoparticles. Fresh mouse blood was collected into tubes with potassium oxalate, centrifuged three times at 3000 *g* for 5 min, and then diluted with PBS at 1:50 v/v. For the hemolytic assay, aqueous UCNP@Ale-P(DMA-AEM)-PEG and UCNP@Ale-P(DMA-AEM)-PEG-mTHPC colloids were diluted with the RBC suspension in a flat bottom 96-well culture plate. After incubation for 60 min at 37 °C, the plate was centrifuged at 3000 *g* for 5 min, and the absorbance of 100 μL of the supernatant was measured at 405 nm using a Tecan Infinite F50 microplate reader. RBCs in PBS were used as a negative control, and RBCs in 0.1% Triton X-100 were used as a positive control. Hemolysis was calculated as follows
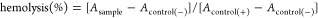
4where *A*_sample_, *A*_control(−)_, and *A*_control(+)_ are the absorbance values at 405 nm of the experimental groups, negative control, and positive control, respectively.

### Pilot *In Vivo* Photodynamic Therapy

PDT was conducted *in vivo* using a cohort of 16 outbred nude female mice (Hsd: athymic Nude-Fox n1nu) with a body weight ranging from 18 to 22 g. The mice, sourced from AnLab and ENVIGO (Prague, Czech Republic), were housed in laminar flow cabinets with radiation-sterilized SAWI bedding from Jelu-Werk (Rosenberg, Germany) and supplied with an irradiated Ssniff diet from Ssniff Spezialdiaeten (Soest, Germany) and unlimited access to autoclaved water.

Ethical approval for all of the experiments was obtained from the ethics committee of the First Faculty of Medicine, Charles University, and the Ministry of Education, Youth, and Sports of the Czech Republic. The experiments adhered to the guidelines set out in Act no. 246/1992 Coll. on the protection of animals against cruelty and Decree 419/2012 on the protection of experimental animals, both in compliance with the legislation of the European Parliament.

Subcutaneously harvested Capan-2 cells (5 × 10^6^) were administered along with BD Matrigel (VWR International; Prague, Czech Republic) into the abdominal right flank of the outbred nude mice. Once the tumors reached a diameter of ∼6 mm, the mice were randomly divided into control and experimental groups (*n* = 4) and underwent ketamine/xylazine narcosis. This was followed by intratumoral application of aqueous UCNP@Ale-P(DMA-AEM)-PEG and UCNP@Ale-P(DMA-AEM)-PEG-mTHPC colloids (100 μL; 1.5 mg/mL). After 10 min, an area of 2 cm^2^ was irradiated for 3 min with a Quanta System IG980 excitation laser (Medicom; Prague, Czech Republic) with a power of 1 W, a power density of 0.5 W/cm^2^, and an energy density of 90 J/cm^2^. Tumor volume and mouse weight were assessed twice a week, and mouse survival was monitored for 30 days.

## Results and Discussion

### Synthesis and Characterization of the UCNP@Ale-P(DMA-AEM)-PEG Colloid

**Figure 1:**
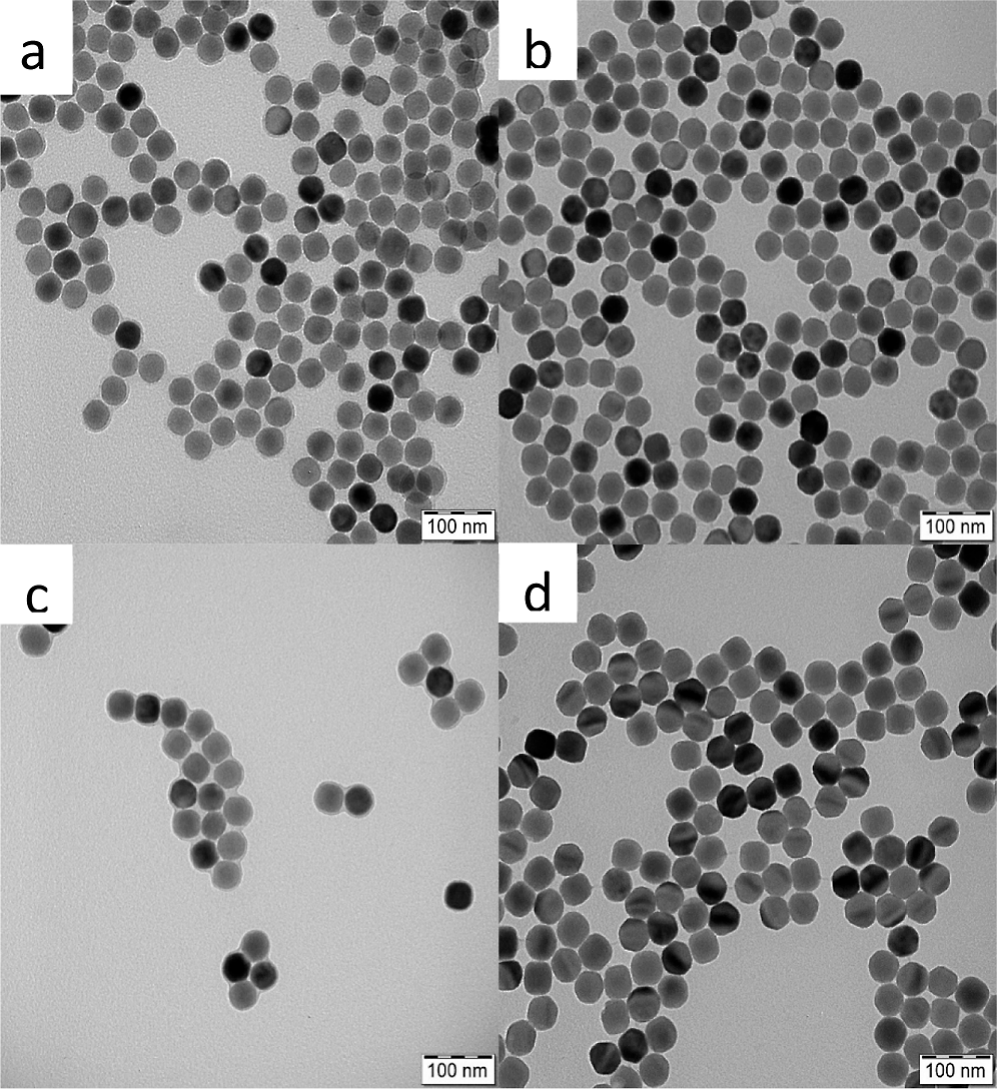
TEM micrographs of the (a) UCNPs, (b) UCNP@Ale-P(DMA-AEM), (c) UCNP@Ale-P(DMA-AEM)-PEG, and (d) UCNP@Ale-P(DMA-AEM)-PEG-mTHPC particles.

It has been previously shown that doping Fe^2+^ ions into conventional NaYF_4_:Yb,Er nanoparticles enhances the upconversion emission in the red region, making the particles suitable for direct excitation of mTHPC as a PDT transducer.^38,40^ The uniformly sized UCNPs were prepared by high-temperature (300 °C) coprecipitation of rare-earth chlorides and iron chloride in octadec-1-ene as a solvent in the presence of oleic acid as a stabilizer. The chemical composition, crystal structure, and upconversion luminescence of the particles were described earlier.^34^ The initial UCNPs stabilized with oleic acid had a spherical shape with *D*_n_ = 39 nm and a narrow size distribution according to TEM (*D̵* = 1.01; Figure 1a and Table 1). This narrow size distribution ensures reproducibility of the results and is critical for consistent physical and biological characteristics. Before surface modification, the hydrophobic UCNPs were washed with hexane, ethanol, and water to remove oleic acid. The polydispersity of the neat UCNPs in water, as measured by DLS, was low (PD = 0.11), although the relatively large *D*_h_ = 191 nm indicated the formation of aggregates (Table 1). The positively charged metal ions on the particle surface then resulted in a positive ζ-potential of the particles (∼43 mV; Table 1).

**Table 1:** TEM and DLS Analyses of Neat and Polymer-Coated UCNPs^a^

particles	*D*_n_ (nm)	*D̵*	*D*_h_ (nm)	PD	ζ-potential (mV)
UCNPs	39	1.01	191 ± 8	0.11	43 ± 3
UCNP@Ale-P (DMA-AEM)	41	1.01	183 ± 6	0.16	33 ± 3
UCNP@Ale-P(DMA-AEM)-PEG	42	1.01	111 ± 3	0.17	10 ± 1
UCNP@Ale-P(DMA-AEM)-PEG-mTHPC	47	1.01	136 ± 3	0.1	22 ± 1

a *D*_n_—number-average diameter (TEM); *D̵*—dispersity (TEM); *D*_h_—hydrodynamic diameter (DLS); PD—polydispersity (DLS).

In this report, poly(*N*,*N*-dimethylacrylamide) was chosen as the main particle coating because of its excellent hydrophilicity and biocompatibility, and it has been successfully used in drug delivery systems.^41^ In addition, polyacrylamide-based polymers have been used to develop third-generation photosensitizers with potent antimicrobial activity for the treatment of primary and metastatic tumors.^42,43^ To introduce reactive amino groups into the polymer with the ability to form complexes with phenolic groups of mTHPC,^44^ DMA was RAFT copolymerized with AEC-Boc. ^1^H NMR spectroscopy confirmed that the polymer contained 10 mol % of the AEC-Boc units (see Supporting Information, Figure S1a). The Ale anchoring groups, which can interact with the metal ions on the particle surface,^45^ were attached to the polymer by carbodiimide chemistry, followed by acidic hydrolysis of the protecting Boc groups. ^31^P and ^1^H NMR spectroscopy confirmed the presence of the Ale group in the P(DMA-AEM)-Ale polymer (δ = 18.4 ppm; Figure S2) and successful deprotection of the amino groups due to the disappearance of the “5” signal (Figure S1b). In the next step, NHS-activated PEG was reacted with the amino groups of UCNP@Ale-P(DMA-AEM) to improve the colloidal stability of the particles. Moreover, the incorporation of PEG into the coating aimed at preventing mTHPC aggregation, and interaction with lipoproteins increased the drug selectivity toward tumors.^24^ Due to its steric hindrance and relatively large size, PEG is assumed to react with the outer amino groups of the P(DMA-AEM) coating, while the remaining groups are available for conjugation with mTHPC. According to the TEM results, the modification of UCNPs by P(DMA-AEM) and PEG had almost no effect on the particle size (*D*_n_ = ∼41 nm) and the size distribution (*D̵* = 1.01; Table 1 and Figure 1b,c). The hydrodynamic diameter of the UCNP@Ale-P(DMA-AEM) particles in water was 183 nm (PD = 0.16), and the ζ-potential was 33 mV (Table 1). With respect to the UCNP@Ale-P(DMA-AEM)-PEG colloid, *D*_h_ decreased to 111 nm with PD PI= 0.17, indicating that the incorporation of PEG improved the colloidal stability of the particles. In contrast to UCNP@Ale-P(DMA-AEM) particles, the PEGylated particles had a lower ζ-potential (10 mV) due to the shielding of the particle charge by electroneutral PEG. The observed shift in the ζ-potential of the PEGylated UCNPs provided further evidence for successful modification of the particle surface.

**Figure 2:**
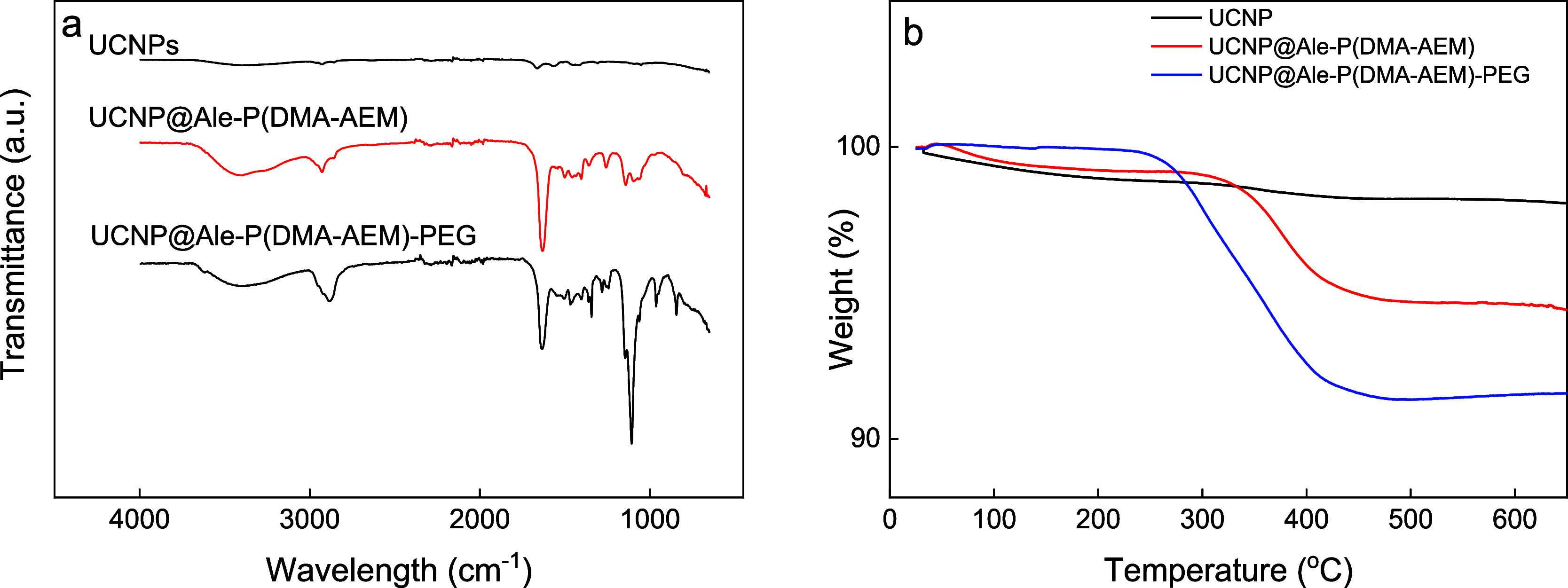
(a) ATR-FTIR spectra and (b) TGA thermograms of UCNPs, UCNP@Ale-P(DMA-AEM), and UCNP@Ale-P(DMA-AEM)-PEG nanoparticles.

In the ATR-FTIR spectrum of the UCNPs (Figure 2a), very weak peaks at 2923, 2856, 1666, and 1565 cm^–1^ were attributed to the asymmetric ν_as_(CH_2_), symmetric ν_s_(CH_3_) and ν(C=C), and asymmetric ν_as_(COO^–^) stretching vibrations of residual oleic acid, respectively.^46^ The FTIR spectrum of the UCNP@Ale-P(DMA-AEM) nanoparticles exhibited a broad peak at 3400 cm^–1^ assigned to the stretching vibrations of ν(NH) and ν(OH), which originated from amino and amide groups and water, respectively. The bands at 2929 and 1632 cm^–1^ were attributed to asymmetric ν_as_(CH_2_) and ν(C=O) stretching vibrations, respectively.^47^ After PEGylation, a new intense peak appeared at 1107 cm^–1^ in the spectrum of the UCNP@Ale-P(DMA-AEM)-PEG particles, which was assigned to ν_s_(−O−) symmetric stretching vibrations of PEG.^48^ According to the TGA thermograms of the UCNP@Ale-P(DMA-AEM) and UCNP@Ale-P(DMA-AEM)-PEG particles, the small weight loss observed during heating from RT to 120 °C was attributed to the desorption of water (Figure 2b). The main decomposition of the polymer on the particle surface was monitored in the range of 220–480 °C. As a result, the UCNP@Ale-P(DMA-AEM) particles had 4.8 wt % coating, and after PEGylation, the amount of coating increased to 8.6 wt %. Thus, FTIR spectroscopy and TGA confirmed the presence of both Ale-P(DMA-AEM) and PEG on the particle surface.

### Chemical and Colloidal Stability of UCNP@Ale-P(DMA-AEM)-PEG Particles

**Figure 3:**
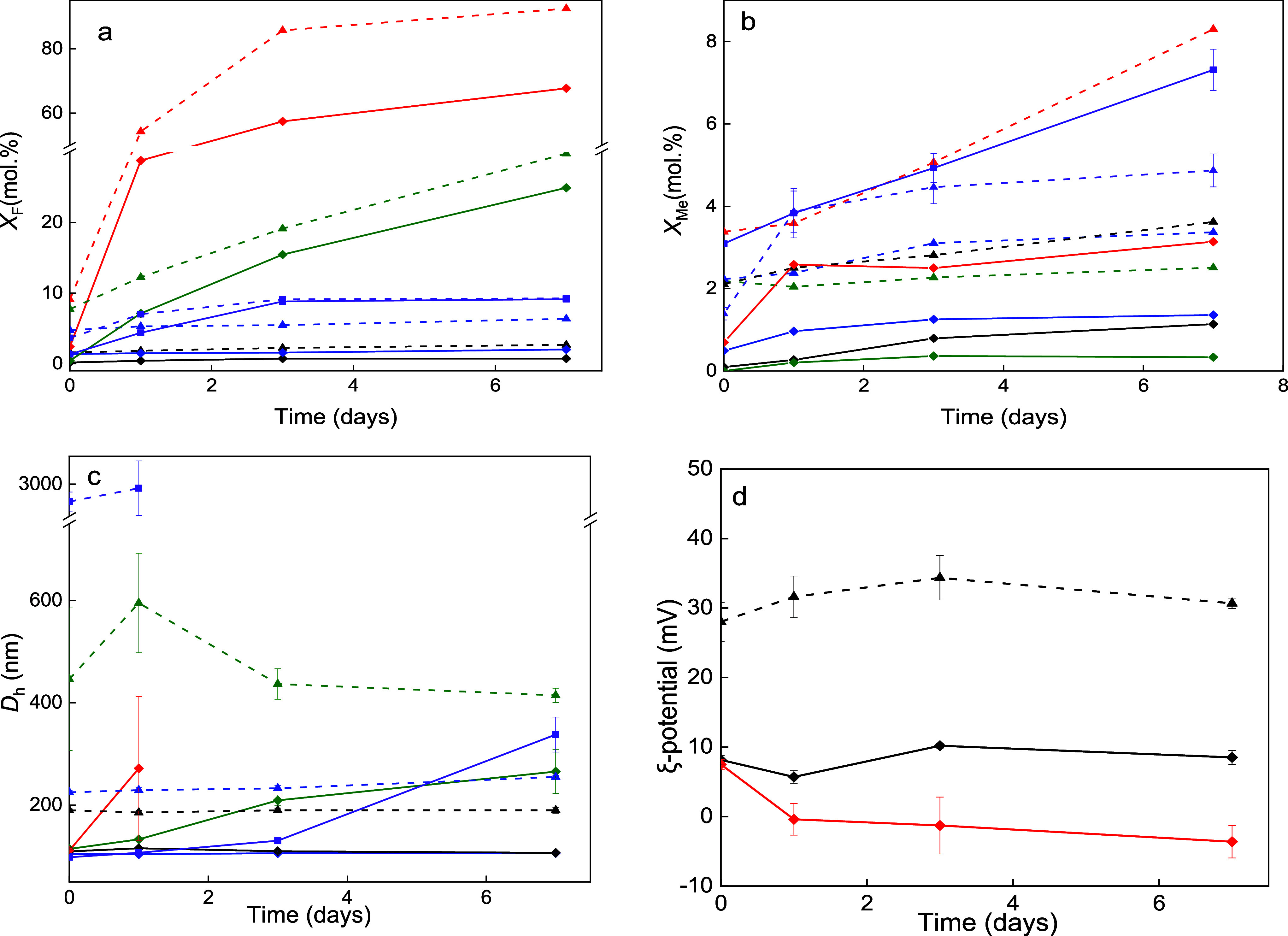
Time dependence of (a) F^–^ (*X*_F_) and the (b) metal ion molar fraction (*X*_Me_) leached from UCNPs (dashed) and UCNP@Ale-P(DMA-AEM)-PEG (solid) in aqueous media (1 mg/mL) at 37 °C. (c) Hydrodynamic diameter *D*_h_ and (d) ζ-potential of the particles in water (black), PBS (red), DMEM (blue), ALF (olive), and AETF (violet). The ζ-potential was determined only in water, DMEM, and PBS because the high salt concentrations in ALF and AETF interfered with the ELS measurements.

An important aspect of the application of UCNPs in PDT is their chemical stability associated with the leaching of toxic fluoride and rare-earth and iron metal ions into the surrounding environment. This leaching reduces the luminescence of the particles and affects the overall performance, genotoxicity, cytotoxicity, binding efficiency, and signal transduction.^49,50^ The rate of particle degradation depends mainly on the temperature and the presence of phosphate ions interacting with metal ions, which significantly accelerates the dissolution of UCNPs.^51,52^ Here, the chemical stability of the UCNPs and UCNP@Ale-P(DMA-AEM)-PEG colloid was evaluated for 7 days by determining the molar fraction of dissolved F^–^ (*X*_F_) and metal ions (*X*_Me_) in commonly used biological media including water, PBS, DMEM, ALF, and AETF at a physiological temperature of 37 °C (Figure 3a,b). Water, PBS, and DMEM are commonly used for *in vivo*/*in vitro* drug screening, while ALF and AETF simulate specific lysosomal and extracellular tumor environments.^36,37^ The release of F^–^ ions from both the UCNPs and UCNP@Ale-P(DMA-AEM)-PEG particles into water was low, reaching 3 and 1 mol %, respectively (Figure 3a). In DMEM, AETF, and ALF, the UCNPs and UCNP@Ale-P(DMA-AEM)-PEG particles also leached relatively little F^–^; the *X*_F_ for neat UCNPs in DMEM, AETF, and ALF was 6, 9, and 30 mol %, respectively, and that for UCNP@Ale-P(DMA-AEM)-PEG was 2, 9, and 25 mol %, respectively (Figure 3a). The dissolution rate of both the neat and coated particles was highest in PBS, releasing up to 93 and 68 mol % F^–^ ions, respectively. Particle degradation in PBS, DMEM, ALF, and AETF was greater than that in water because these media contained 10, 0.9, 0.5, and 1.47 mM phosphate ions, respectively. However, PBS affected chemical stability the most, as it had the highest content of these ions among these media. Although DMEM contains more phosphates than ALF does, the *X*_F_ of particles in DMEM is much lower than that in ALF due to the formation of a protein corona that protects the particles from degradation.^53^ These results thus suggest that the nanoparticles will be dissolved in the cells predominantly inside the lysosomes. In contrast to the neat UCNPs, the Ale-P(DMA-AEM)-PEG coating provided a relatively good protection against particle dissolution. The leakage of F^–^ into water, PBS, DMEM, ALF, and AETF at 37 °C for 7 days was reduced by 74, 27, 68, 16, and 3%, respectively. As far as metal ion leaching from UCNPs in PBS is concerned, only 8% of the metal ions released were found by UV–vis spectroscopy (Figure 3b). The low amount of rare-earth atoms relative to the high amount of dissolved fluorine can be explained by the precipitation of metal ions by disodium hydrogen phosphate, which results in phosphates being known to have very low water solubility (∼10^–13^ M).^54^ The leaching of metal ions from the uncoated UCNPs in water, ALF, AETF, and DMEM medium reached 3.6, 2.5, 4.9, and 3.4 mol %, respectively. In contrast, incubating UCNP@Ale-P(DMA-AEM)-PEG particles in water, PBS, DMEM, or ALF for at least 7 days did not cause any significant leaching of metal ions, which amounted to 1.2, 3.1, 1.4, and 0.3 mol %, respectively. Exposure of the particles to AETF at 37 °C for 7 days induced a more pronounced leaching of metal ions (7.3 mol %). This suggests that these particles may release these ions in the acidic microenvironment of tumor tissues, which may contribute to their destruction. Thus, the release of metal ions from both neat and polymer-coated UCNPs was the lowest in the ALF.

One of the main challenges of designing UCNPs for PDT is maintaining their dispersibility in aqueous media and preventing aggregation, which decreases the mTHPC binding efficiency, quenches fluorescence, and reduces ROS generation and treatment efficiency. Hence, the colloidal stability of the UCNPs and UCNP@Ale-P(DMA-AEM)-PEG particles incubated in water and relevant biological media at 37 °C was determined via DLS (Figure 3c). The neat UCNPs were stable in DMEM and water (*D*_h_ = 240 ± 15 and 190 ± 5 nm, respectively), and their ζ-potential in water was ∼30 mV due to the presence of metal ions on the surface (Figure 3d). In contrast to that in water, the *D*_h_ values of the UCNPs in ALF increased after 7 days from 190 to ∼500 nm, indicating a tendency to aggregate. In PBS and AETF, the particles immediately aggregated (*D*_h_ > 1 μm) due to the formation of a counterion layer. Compared to those of the neat UCNPs, the UCNP@Ale-P(DMA-AEM)-PEG particles had better colloidal stability. The size of the particles in water and DMEM was ∼111 nm, and the size remained constant for 7 days without any sign of particle sedimentation. In ALF, the *D*_h_ increased from 111 to 265 nm after 7 days of incubation, suggesting the formation of particle assemblies, probably due to partial exchange of the polymer coating with phosphates.^55^ AETF showed similar behavior, where *D*_h_ increased from 98 to 130 nm after 3 days of incubation and reached 338 nm after 7 days. The *D*_h_ of the UCNP@Ale-P(DMA-AEM)-PEG particles in PBS increased from 111 to 270 nm within 24 h, and aggregation (*D*_h_ > 1 μm) occurred after 3 days due to phosphate replacement of the coating. The ζ-potential of the UCNP@Ale-P(DMA-AEM)-PEG particles in water was 10 mV, which decreased to zero in PBS due to the presence of the counterion layer (Figure 3d). The results thus demonstrated that particles can be exposed to PBS for a short time without significant deterioration of their properties, which is important for their intratumoral administration. The relatively good colloidal stability in AETF, which mimics the tumor microenvironment, can ensure long-term particle interactions with tumor cells, which could enhance the efficacy of PDT. Furthermore, the UCNP@Ale-P(DMA-AEM)-PEG particles exhibited excellent dispersibility and colloidal stability in aqueous culture media (DMEM), maintaining their intracellular functionality for at least 7 days, which is important for PDT treatment using photosensitizer-conjugated UCNPs.

### Conjugation of mTHPC to UCNP@Ale-P(DMA-AEM)-PEG Particles

The attachment of mTHPC to a polymer on a UCNP surface is an appropriate approach for overcoming photosensitizer aggregation and release from the carrier, which can reduce the treatment efficacy. After introducing mTHPC into the polymer shell of UCNP@Ale-P(DMA-AEM)-PEG particles, *D*_n_ and *D̵* hardly changed, while the ζ-potential and *D*_h_ in water increased slightly to 22 mV and 136 nm, respectively, indirectly confirming the conjugation (Figure 1d; Table 1). The UV–vis spectrum of the UCNP@Ale-P(DMA-AEM)-PEG-mTHPC colloid showed an intense Soret band in the blue region and Q-bands in the 500–680 nm region, which are typical of temoporfin^33^ and provide evidence of its presence in UCNPs (Figure S3). The amount of bound mTHPC was 0.4 μg/mg of UCNPs.

**Figure 4:**
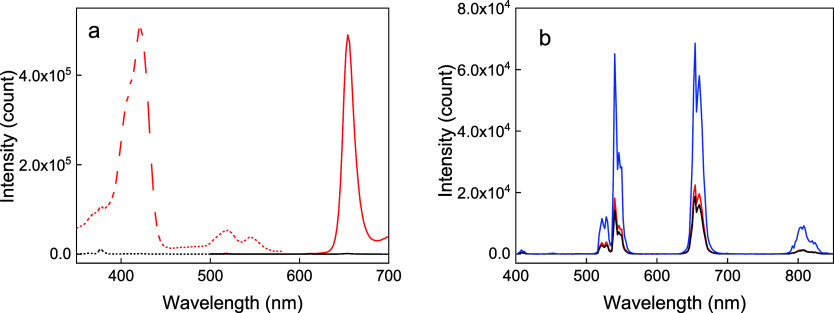
(a) Excitation (dashed; λ_em_ = 652 nm), emission (solid; λ_ex_ = 422 nm), and (b) upconversion emission spectra of UCNPs (blue), UCNP@Ale-P(DMA-AEM)-PEG (black), and UCNP@Ale-P(DMA-AEM)-PEG-mTHPC particles (red) in water (1 mg/mL) at 980 nm excitation and a laser power density of 2.11 W/cm^2^.

Conjugation of mTHPC to UCNP@Ale-P(DMA-AEM)-PEG particles provided luminescence at 654 nm and corresponding excitation at 420 nm, whereas UCNPs without mTHPC did not show any characteristic photoluminescence peaks characteristic of temoporfin (Figure 4a). Bands at 409 nm (^2^H_9/2_ → ^4^I_15/2_), 525 nm (^2^H_11/2_ → ^4^I_15/2_), 542 nm (^4^S_3/2_ → ^4^I_15/2_), 656 nm (^4^F_9/2_ → ^4^I_15/2_), and 807 nm (^4^I_9/2_ → ^4^I_15/2_), corresponding to characteristic Er^3+^ emission transitions, were observed in the upconversion photoluminescence emission spectra of both the uncoated and polymer-coated UCNP aqueous dispersions excited at 980 nm (Figure 4b). Modification of UCNPs with Ale-P(DMA-AEM)-PEG and binding of mTHPC quenched the upconversion emission due to inhomogeneities in the polymer coating and water penetration to the particle surface.

### ROS Generation

**Figure 5:**
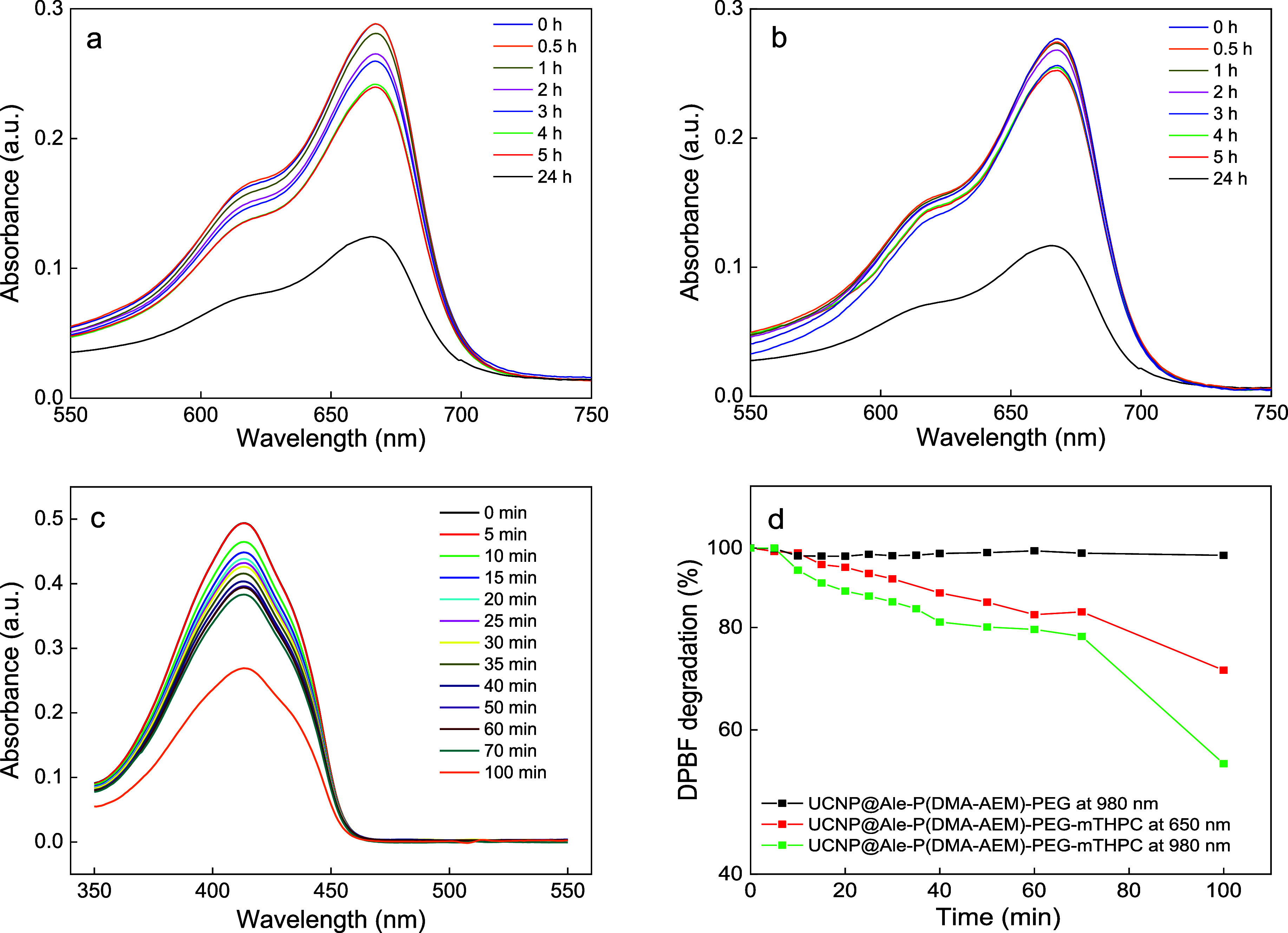
UV–vis spectra documenting the time dependence of (a,b) methylene blue degradation in (a) water and (b) PBS in the presence of UCNP@Ale-P(DMA-AEM)-PEG particles (2 mg/mL) and H_2_O_2_. (c) DPBF degradation in ethanol/H_2_O (50:50 v/v) containing UCNP@Ale-P(DMA-AEM)-PEG-mTHPC particles (2 mg/mL) *versus* irradiation time at 980 nm excitation with a power density of 2.11 W/cm^2^. (d) Degradation rate of DPBF at 415 nm in the presence of UCNP@Ale-P(DMA-AEM)-PEG-mTHPC particles upon 650 nm (150 W xenon lamp) and 980 nm laser irradiation (2.11 W/cm^2^). Particles without mTHPC were used as a control.

Iron-containing upconversion nanoparticles are known to catalyze the Fenton reaction, triggering the conversion of intracellular H_2_O_2_ into highly damaging hydroxyl radicals, thus enhancing PDT efficacy and killing tumors.^34,56^ In PDT, the generation of hydroxyl radicals is beneficial for overcoming tumor hypoxia and inducing ferroptosis in tumor cells.^57,58^ The generation of hydroxyl radicals by autoxidation of Fe^2+^ ions accompanied by oxidative degradation of methylene blue due to the reaction of the UCNP@Ale-P(DMA-AEM)-PEG colloid with H_2_O_2_ was monitored spectrophotometrically (Figure 5a,b). UV–vis spectra of the particle dispersions in both water and PBS showed a time-dependent decrease in the methylene blue absorption intensity at 666 nm. The generation of hydroxyl radicals in aqueous media was accompanied by good colloidal stability of the particles, which facilitated the penetration of the solution to the particle surface and the Fenton reaction. In PBS, radical formation was attributed to more pronounced ion leaching than in water, which was confirmed by a stability study.

Singlet oxygen generation from the UCNP@Ale-P(DMA-AEM)-PEG-mTHPC colloid was determined after irradiation with a 980 nm NIR laser using a DPBF probe. Exposure of a DPBF solution in an ethanol/water mixture containing mTHPC-conjugated particles to NIR light for 100 min caused photobleaching of DPBF (Figure 5c,d). The decrease in the DPBF absorbance at 415 nm with increasing irradiation time indicated efficient energy transfer from the excited particles to the photosensitizer and increased singlet oxygen production. The UCNP@Ale-P(DMA-AEM)-PEG-mTHPC and UCNP@Ale-P(DMA-AEM)-PEG particles were also tested under the same conditions when irradiated with a 150 W xenon lamp at 650 nm. The time-dependent decrease in the DPBF absorbance of UCNP@Ale-P(DMA-AEM)-PEG-mTHPC correlated perfectly with the observed DPBF degradation rate after irradiation at 980 nm (Figure 5d). The lower degradation rate observed for 650 nm light was due to the differences in power and irradiation area between the xenon lamp and the NIR laser. Moreover, the UCNP@Ale-P(DMA-AEM)-PEG colloid (without mTHPC) showed no DPBF absorbance after laser exposure (Figure 5d). This confirmed that the degradation rate of DPBF was induced by efficient energy transfer from UCNPs to mTHPC, resulting in ROS generation.

**Figure 6:**
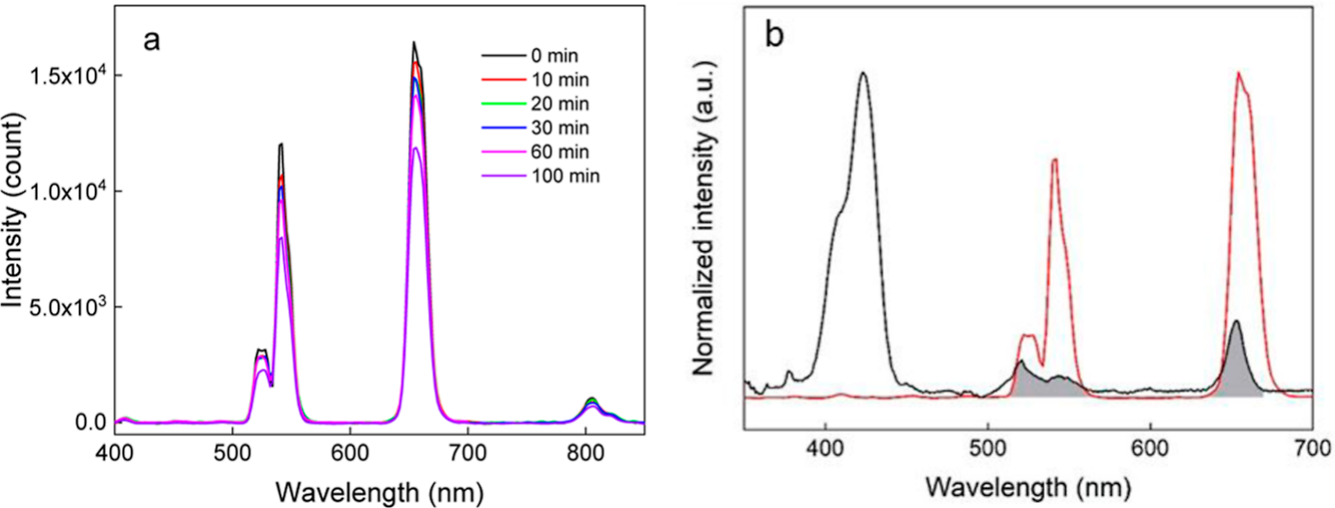
(a) Upconversion photoluminescence emission spectra of DPBF in ethanol/H_2_O (50:50 v/v) containing UCNP@Ale-P(DMA-AEM)-PEG-mTHPC particles (2 mg/mL) versus irradiation time at 980 nm excitation with a power density of 2.11 W/cm^2^. (b) Overlap (gray) of the absorption spectrum of mTHPC (black) and emission spectrum of the upconversion luminescence of the UCNPs (red) at 980 nm excitation.

As expected, ROS generation confirmed efficient energy transfer from UCNPs to mTHPC, which was also supported by photoluminescence upconversion emission spectra after 980 nm excitation, demonstrating an obvious decrease in luminescence intensity at 525, 542, and 656 nm with increasing irradiation time (Figure 6a). This is consistent with the overlapping spectra between the upconversion emission of UCNPs and the absorption Q-bands of mTHPC in the 500–680 nm region (Figure 6b).

### *In Vitro* Biocompatibility of UCNP@Ale-P(DMA-AEM)-PEG-mTHPC Particles

**Figure 7:**
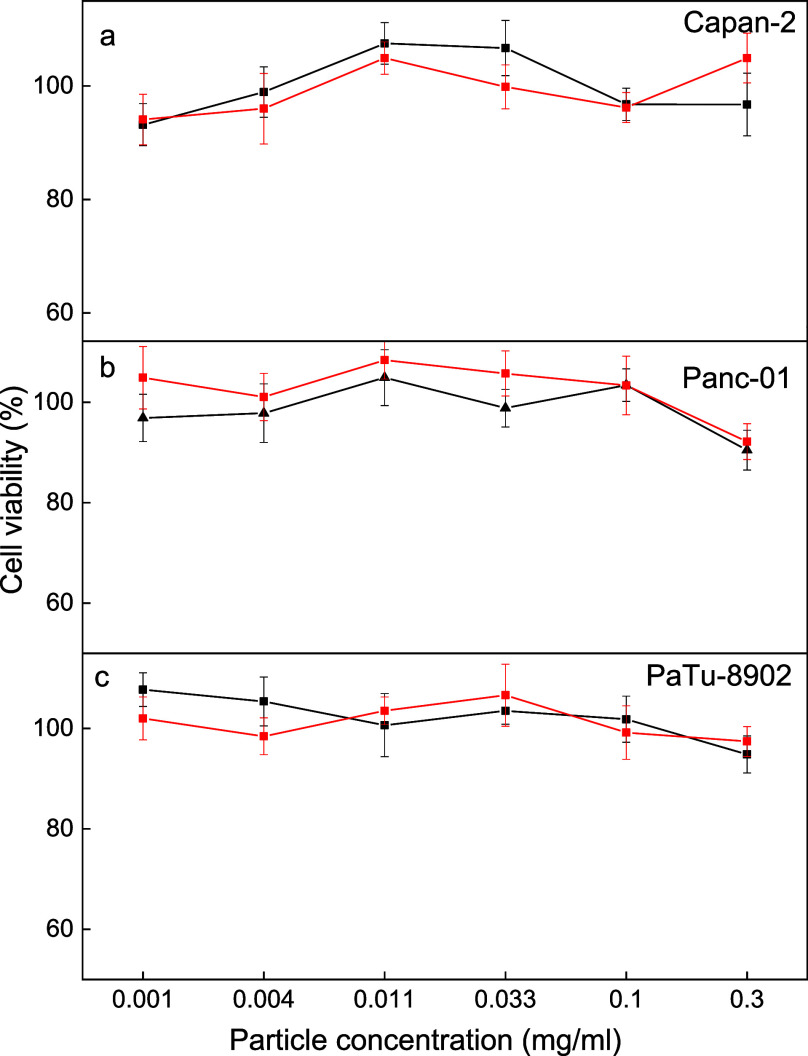
*In vitro* dark cytotoxicity (without irradiation) of UCNP@Ale-P(DMA-AEM)-PEG (black) and UCNP@Ale-P(DMA-AEM)-PEG-mTHPC colloids (red) against (a) Capan-2, (b) PANC-1, and (c) PaTu-8902 human pancreatic adenocarcinoma cell lines according to the XTT assay.

Before the UCNP@Ale-P(DMA-AEM)-PEG-mTHPC particles were used in NIR-induced PDT *in vivo*, it was necessary to test their cytotoxicity to ensure the safety of this delivery system. Therefore, the dark toxicity of UCNP@Ale-P(DMA-AEM)-PEG and UCNP@Ale-P(DMA-AEM)-PEG-mTHPC colloids was tested without 980 nm irradiation. For this task, Capan-2, PANC-1, and PA-TU-8902 human pancreatic adenocarcinoma cell lines were incubated with the nanoparticles in the concentration range of 0–0.3 mg/mL for 48 h, and a standard XTT assay was used to evaluate the cytotoxic potential of the colloids. None of these cell lines exhibited a statistically significant decrease in the viability after incubation with either type of colloid (Figure 7). It can thus be concluded that neither UCNP@Ale-P(DMA-AEM)-PEG nor UCNP@Ale-P(DMA-AEM)-PEG-mTHPC colloids were harmful to these cells, even at a maximum concentration of 0.3 mg/mL. Such a high concentration corresponds to a potential dose of 1.5 g of particles in the whole blood of the animal. In addition, the concentrations used were similar to those previously published for UCNPs containing mTHPC.^33^ This may qualify the use of the UCNP@Ale-P(DMA-AEM)-PEG-mTHPC colloid in PDT.

**Figure 8:**
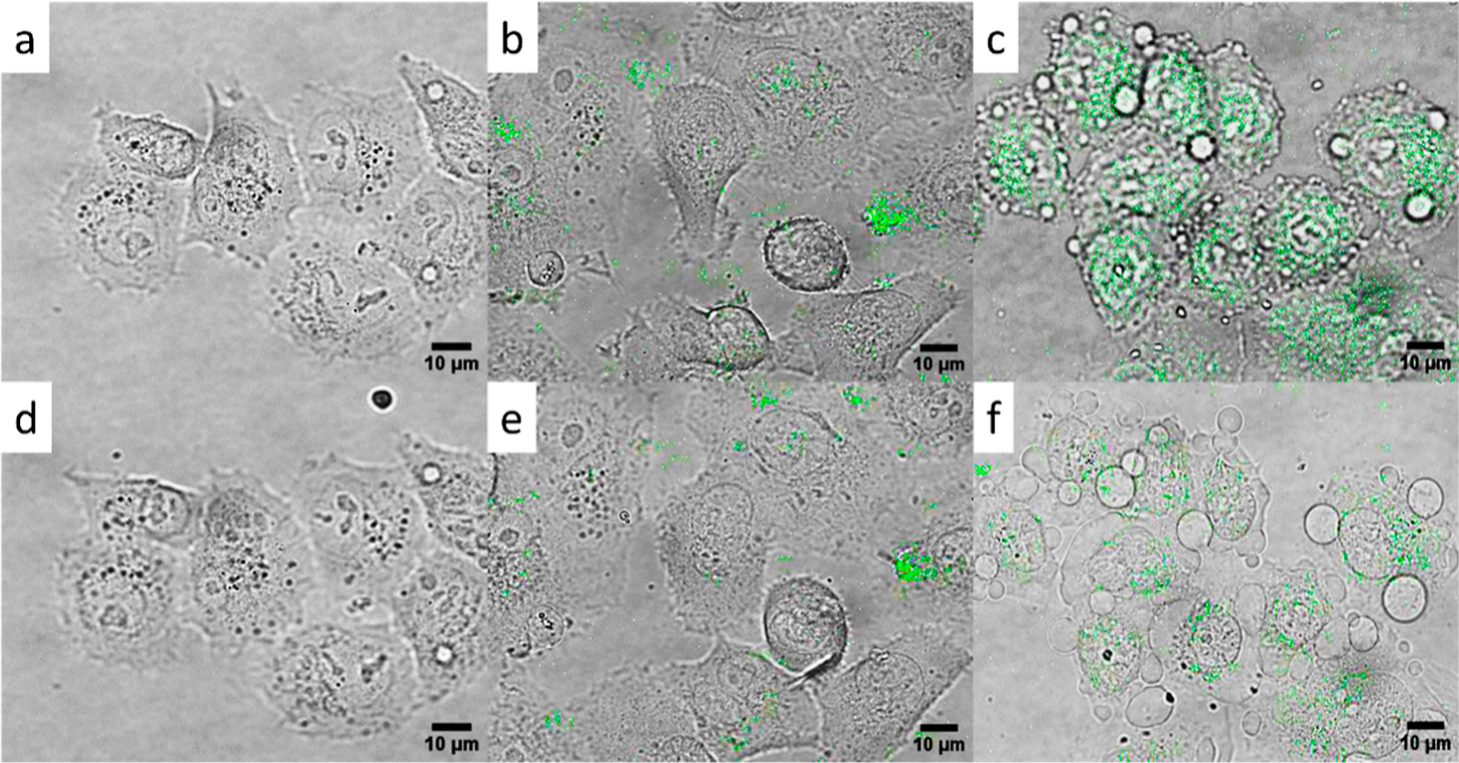
*In vitro* PDT experiment: overlays of bright-field micrographs and upconversion photoluminescence after excitation at 980 nm. (a,d) Control PANC-1 cells without UCNPs, (b,e) cells with UCNP@Ale-P(DMA-AEM)-PEG and (c,f) with UCNP@Ale-P(DMA-AEM)-PEG-mTHPC particles (green) after (a–c) 0 and (d–f) 30 min of irradiation.

**Figure 9:**
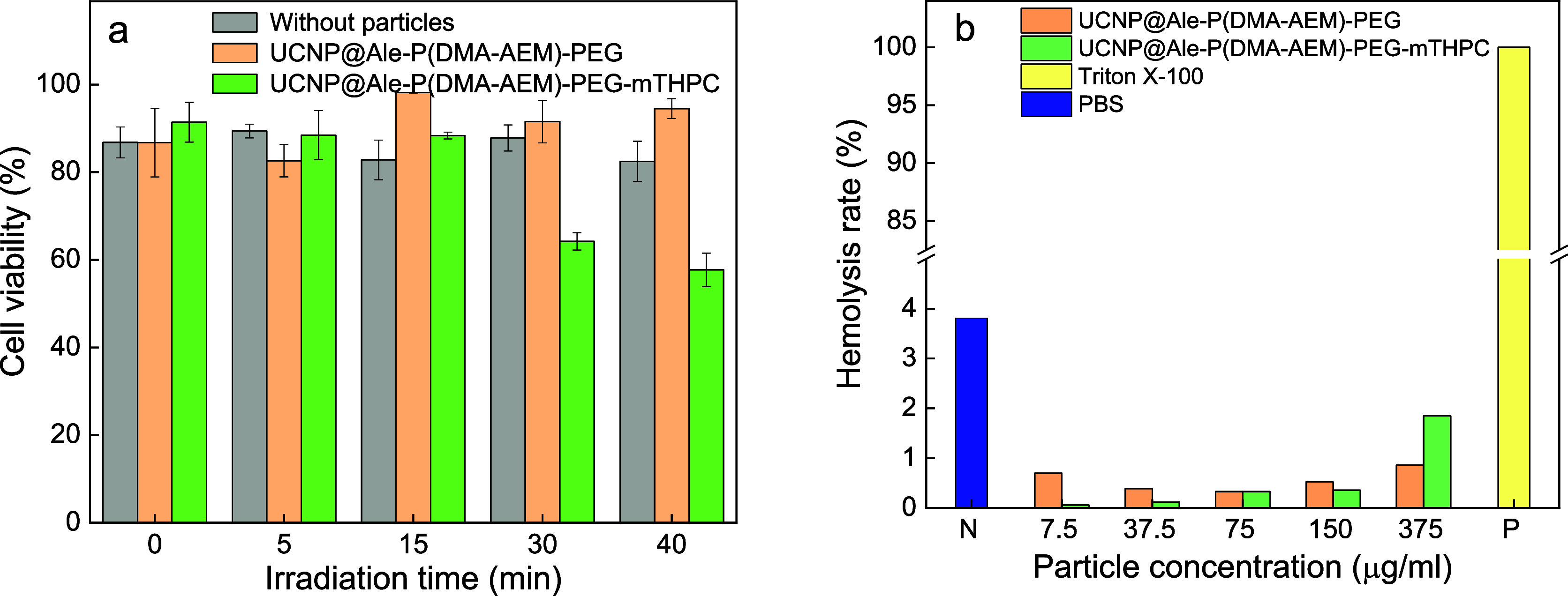
(a) *In vitro* cytotoxicity of UCNP@Ale-P(DMA-AEM)-PEG and UCNP@Ale-P(DMA-AEM)-PEG-mTHPC particles against human pancreatic adenocarcinoma cell line PANC-1 after irradiation with 980 nm laser (0.7 W/cm^2^) for different time periods. (b) Hemolysis rate of RBCs treated with PBS (N; negative control), Triton X-100 (P; positive control), and different concentrations of UCNP@Ale-P(DMA-AEM)-PEG and UCNP@Ale-P(DMA-AEM)-PEG-mTHPC particles.

The photodynamic effect of UCNP@Ale-P(DMA-AEM)-PEG-mTHPC particles was investigated in model pancreatic cancer PANC-1 cells after irradiation with 980 nm laser. The cells were incubated with both UCNP@Ale-P(DMA-AEM)-PEG (control) and UCNP@Ale-P(DMA-AEM)-PEG-mTHPC particles; cells without nanoparticles served as an additional control. The particles were nicely distributed in the cell cytoplasm but not in the nucleus (Figure 8). After laser irradiation for 30 min, cells containing UCNP@Ale-P(DMA-AEM)-PEG-mTHPC particles were destroyed (Figure 8c,f) due to singlet oxygen generation, in contrast to cells incubated with UCNP@Ale-P(DMA-AEM)-PEG particles without mTHPC (Figure 8b,e) and cells containing no particles (Figure 8a,d). Figure 8a,d,b,e thus confirmed that the laser irradiation alone did not damage the cells. The data were also comparable to the in vitro cytotoxicity of UCNP@Ale-P(DMA-AEM)-PEG and UCNP@Ale-P(DMA-AEM)-PEG-mTHPC colloids against human pancreatic adenocarcinoma PANC-1 cell lines irradiated with 980 nm laser (0.7 W/cm^2^) for various time durations (Figure 9a). No decrease in viability was observed after incubation with nanoparticles without mTHPC after 40 min of laser irradiation, whereas UCNP@Ale-P(DMA-AEM)-PEG-mTHPC particles were toxic after laser exposure, with cell viability ∼50%. This demonstrated the generation of ROS and thus the applicability of UCNP@Ale-P(DMA-AEM)-PEG-mTHPC particles for the PDT of tumors.

It was also important to investigate the biocompatibility of the colloids with blood cells in vitro in order to predict long-term interactions of the particles *in vivo* with soft tissues. Hemolysis in the presence of UCNP@Ale-P(DMA-AEM)-PEG and UCNP@Ale-P(DMA-AEM)-PEG-mTHPC particles *in vitro* showed no adverse reactions (Figure 9b). The hemolysis rate at different particle concentrations was <5% and comparable to the negative control (PBS). Thus, the UCNP@Ale-P(DMA-AEM)-PEG-mTHPC colloid showed good biocompatibility without damaging red blood cells.

### *In Vivo* NIR-Induced PDT of Pancreatic Adenocarcinoma in an Animal Model with the UCNP@Ale-P(DMA-AEM)-PEG-mTHPC Colloid

**Figure 10:**
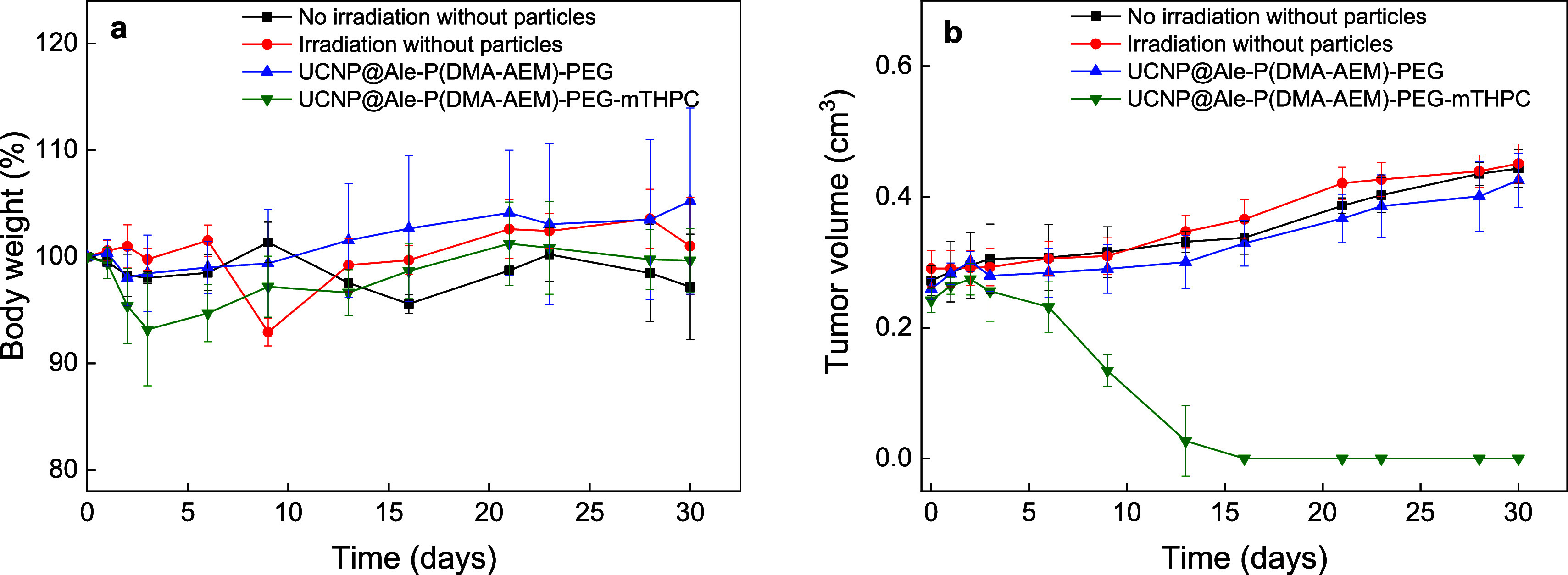
Pilot *in vivo* NIR-induced PDT of subcutaneously growing Capan-2 pancreatic tumors with the UCNP@Ale-P(DMA-AEM)-PEG-mTHPC colloid. Time dependence of (a) nu/nu mouse weight (systemic toxicity) and (b) tumor volume growth in mice (*n* = 4) intratumorally injected with 100 μL of PBS (two controls, without and with irradiation) and UCNP@Ale-P(DMA-AEM)-PEG or UCNP@Ale-P(DMA-AEM)-PEG-mTHPC particles in PBS (100 μL; 1.5 mg/mL); 10 min after administration, the mice were irradiated with a 980 nm laser (0.5 W/cm^2^) for 3 min. The difference between UCNP@Ale-P(DMA-AEM)-PEG-mTHPC and the other three groups was statistically significant (*p* < 0.01).

Although the UCNP@Ale-P(DMA-AEM)-PEG-mTHPC colloid did not cause any dark toxicity *in vitro* as mentioned above, its potential *in vivo* therapeutic efficacy was evaluated in a 30 day pilot study in immunodeficient nude mice subcutaneously injected with the human pancreatic adenocarcinoma Capan-2. Mouse weights were regularly monitored, and body weight changes were compared between the experimental groups to detect any signs of systemic toxicity manifested by body weight loss. In accordance with the *in vitro* findings, no signs of toxicity or adverse effects were observed *in vivo* (Figure 10a), indicating that the UCNP@Ale-P(DMA-AEM)-PEG-mTHPC nanoparticles were safe for administration to animals.

**Figure 11:**
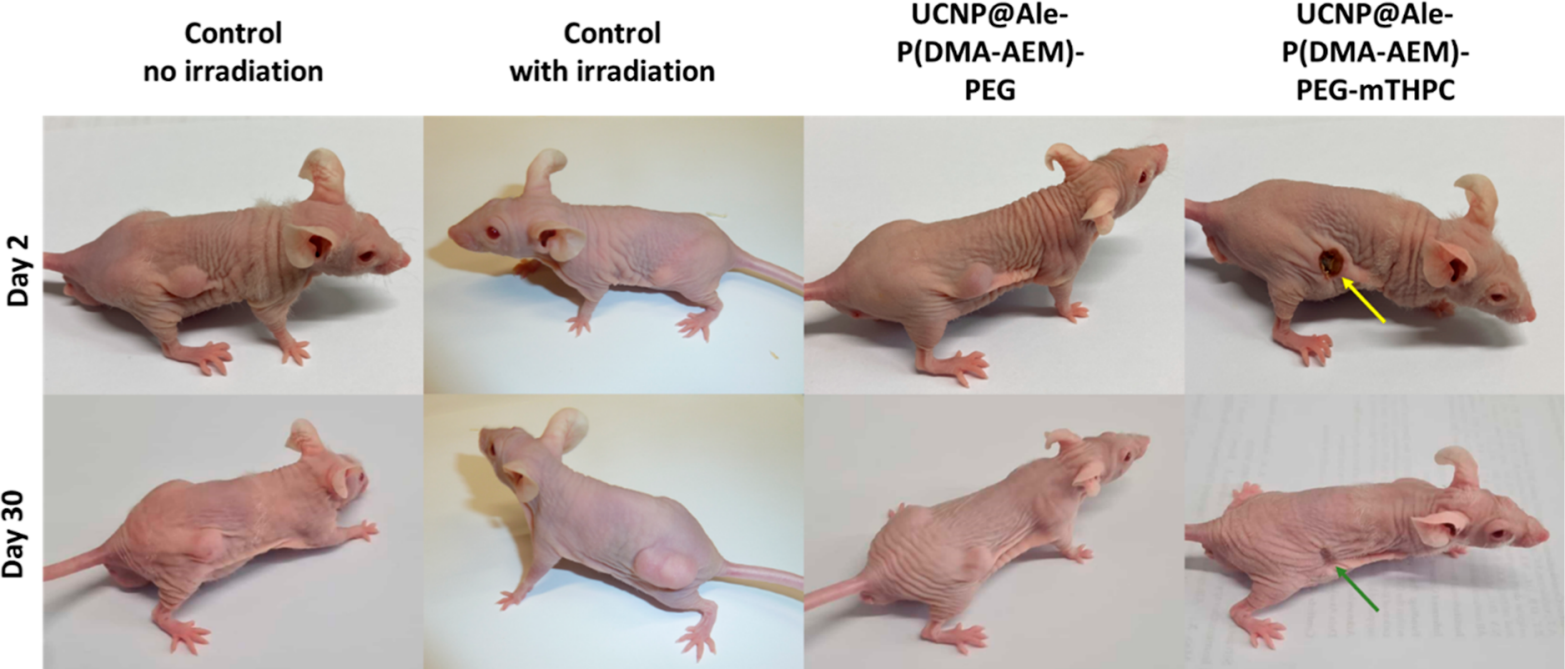
Nu/nu mice with growing human pancreatic adenocarcinoma Capan-2 two (top line) and 30 days (bottom line) after 980 nm NIR-triggered PDT (0.5 W/cm^2^) for 3 min. Yellow arrow, necrosis in the UCNP@Ale-P(DMA-AEM)-PEG-mTHPC-treated group; green arrow, scar after tumor healing. Controls: untreated mice were given an intratumoral injection of PBS, without and with irradiation.

Before *in vivo* NIR-induced PDT treatment of pancreatic adenocarcinoma, nude mice with subcutaneously growing human Capan-2 pancreatic adenocarcinomas were randomly divided into 4 groups: a control group without irradiation, a control group without administered colloid and with irradiation, a group with UCNP@Ale-P(DMA-AEM)-PEG colloid (without THPC) and irradiation, and a group with UCNP@Ale-P(DMA-AEM)-PEG-mTHPC colloid and irradiation. Individual groups of mice were intratumorally injected with PBS or colloids, and after 10 min, the mice were irradiated with 980 nm light for 3 min at a biosafe power density of 500 mW/cm^2^. No further irradiation was performed during the follow-up phase. While no reaction was observed in the three groups without mTHPC (Figure 10b), mice treated with the UCNP@Ale-P(DMA-AEM)-PEG-mTHPC colloid developed extensive necrosis within 1–2 days after PDT (Figures 11 and S4). All four mice in this group exhibited complete remission of cancer (Figure 10b), and the necrosis completely healed, resulting in a visible scar on the skin (Figure S4).

Moreover, in the concurrent control experiment, the UCNP@Ale-P(DMA-AEM)-PEG colloid without THPC was administered to mice under identical conditions, and tumor growth was not affected compared to that of the PBS-treated controls with or without irradiation. Notably, various types of clinically approved PDT photosensitizers in combination with UCNPs irradiated at 980 nm have been investigated in the literature for the treatment of many kinds of cancer (Table 2). Several studies have used power densities higher than the safety threshold of 0.72 W/cm^2^. Most related works have used long exposure times and described only the overall efficacy of these treatments in tumor suppression. However, the relatively low energy conversion and poor photosensitizer loading were the main reasons for the low PDT efficacy of the UCNPs, which further limited their application in *in vivo* tumor treatment.^59^ An advantage of our pilot *in vivo* NIR-induced PDT study is that it demonstrated the great potential of the mTHPC-conjugated UCNP@Ale-P(DMA-AEM)-PEG colloid in the treatment of pancreatic adenocarcinoma. However, further research is needed on this highly promising colloid, focusing on its *in vivo* biodistribution profile after intravenous and/or intraperitoneal administration and testing its efficacy against different tumor types.

**Table 2:** *In Vivo* Performance of Clinically Approved Photosensitizers Studied in 980 nm NIR-Induced UCNP-Based PDT

photosensitizer	particles	coatings	tumor cells	route of administration	laser power (mW/cm^2^)	irradiation time (min)	PDT effect	references
methylene blue	NaYF_4_:Yb^3+^,Er^3+^	SiO_2_–Au nanorods-folic acid	OECM-1 oral	intratumoral	200	30	tumor suppression	(60)
pyropheophorbide-*a*	NaYF_4_:Yb^3+^,Er^3+^	polyethylenimine-*O*-carboxymethyl chitosan-(RGDyK)peptide	U87-MG brain	intravenous	500	30	tumor suppression	(61)
5-aminolevulinic acid (protoporphyrin IX precursor)	NaErF_4_:Tm^3+^@NaYF_4_	poly(ethylene glycol)-folic acid	MCF-7 breast	intratumoral	636	20	tumor suppression	(62)
	NaYF_4_:Yb^3+^,Er^3+^@CaF_2_	poly(acrylic acid)-hydrazide	4T1 breast	intratumoral	500	40	tumor suppression	(63)
chlorin-e6	NaYF_4_:Yb^3+^,Er^3+^	poly(ethylene glycol)	4T1 breast	intratumoral	500	30	partial remission	(64)
	NaYF_4_:Yb^3+^,Er^3+^,Mn^2+^	SiO_2_-hydrocarbonoctadecyl-trimethoxysilane-poly(ethylene glycol)-3-{[10-[3-(methacryloyloxy)propoxy] anthracen-9-yl]oxy} propylstearate	KB oral	intratumoral	500	30	tumor suppression	(65)
	NaYF_4_:Yb^3+^,Er^3+^,Mn^2+^	poly(acrylic acid) adsorbed two layer of poly(allylamine hydrochloride) and dimethylmaleic acid-polyethylene glycol layer	4T1 breast	intratumoral	500	300	tumor suppression	(66)
	NaYF_4_:Yb^3+^,Er^3+^@NaGdF_4_	polyamidoamine-catalase-(3-carboxypropyl) triphenylphosphonium bromide	4T1 breast	intravenous	500	10	tumor suppression	(67)
	NaYF_4_:Yb^3+^,Er^3+^@NaGdF_4_	poly(ethylene glycol)-phospholipid	U87-MG brain	intravenous	600	5	tumor suppression	(68)
chlorin-e6-Mn^2+^ complex	NaScF_4_:Yb^3+^,Er^3+^@CaF_2_	poly(acrylic acid)-human serum albumin	U87 brain	intravenous	1500	30^b^	tumor suppression	(69)
temoporfin^a^	NaYF_4_:Yb^3+^,Er^3+^	cholesterol-poly(ethylene glycol)-angiopep-2	ALTS1C1 brain	intravenous	800^c^	5	life extension	(32)
temoporfin	NaYF_4_:Yb^3+^,Er^3+^,Fe^2+^	poly(methyl vinyl ether-*alt*-maleic acid)	Capan-2 pancreatic	intratumoral	500	3	tumor suppression^d^	(34)
		poly[*N*,*N*-dimethylacrylamide-*co*-2-aminoethylacrylamide]-*graft*-poly(ethylene glycol)-alendronate	Capan-2 pancreatic	intratumoral	500	3	complete remission	this work

a Combined with IR-780 dye.

b Every 24 h for 14% days.

c Combined with 808 nm laser (0.36 W/cm^2^; 3 min).

d Combined with chemotherapy effect of IR-780 dye.

## Conclusions

Due to its noninvasive nature and spatiotemporal precision, NIR-induced PDT is an effective strategy for localized, precise, and deep tissue penetrating treatment. This therapy can overcome the potential risks of treating various deep-seated tumors and the spread of tumor cells by increasing in situ pressure.^70^ In this work, a novel water-dispersible mTHPC-conjugated UCNP@Ale-P(DMA-AEM)-PEG colloid was successfully developed for 980 nm NIR-induced PDT of pancreatic cancer, which contributes significantly to the mortality rate of the human population. The presence of Fe^2+^ ions in the UCNPs enabled the production of hydroxyl radicals to achieve highly efficient PDT. Modification of Ale-P(DMA-AEM) with PEG improved the colloidal and chemical stability of the particles in physiological fluids and thus improved therapeutic efficacy. The upconversion emission produced by these UCNPs efficiently activated conjugated temoporfin at different wavelengths, generating singlet oxygen. This unique emission profile also provides an interesting means for *in vivo* imaging and tracking, which may be useful in future image-guided therapies. A pilot *in vivo* experiment with NIR-triggered PDT demonstrated complete tumor regression of human pancreatic adenocarcinoma growing subcutaneously in athymic mice. The excellent *in vivo* results obtained in mice may open new avenues for the noninvasive therapy of human pancreatic cancer in the future. The combination of ferroptosis and PDT with the UCNP@Ale-P(DMA-AEM)-PEG-mTHPC colloid can compensate for the lack of efficacy of ferroptosis alone and reduce tumor hypoxia. Thus, the results of this study on the development of a novel mTHPC-conjugated UCNP@Ale-P(DMA-AEM)-PEG colloid might be very useful in clinical practice for PDT of cancer under low NIR exposure.

## Supporting Information Available

^1^H and ^31^P NMR spectra of polymers, UV–vis spectra of particles, and micrographs of nu/nu mice with growing human Capan-2 pancreatic adenocarcinoma treated with the particles (PDF)
