# Supplementary material for: Temoporfin-Conjugated PEGylated Poly(N,N-dimethylacrylamide)-Coated Upconversion Colloid for NIR-Induced Photodynamic Therapy of Pancreatic Cancer
Source: Biomacromolecules. 2024 Jun 18;25(9):5771–85. doi: 10.1021/acs.biomac.4c00317 (PMC11388470; doi:10.1021/acs.biomac.4c00317)
Supplement: Supplementary file 1 — bm4c00317_si_001.pdf [file bm4c00317_si_001.pdf]

Supporting Information

Temoporfin-conjugated PEGylated poly(*N,N*-dimethylacrylamide)-coated upconversion colloid for NIR-induced photodynamic therapy of pancreatic cancer

Oleksandr Shapoval[1,*], *Vitalii Patsula*[1], *David Větvička*[2], *Hana Engstová*[3], *Viktoriia Oleksa*[1], *Martina Kabešová*[2], *Taras Vasylyshyn*[1], *Pavla Poučková*[2], *Daniel Horák*[1,*]

[1] Institute of Macromolecular Chemistry, Czech Academy of Sciences, Heyrovského nám. 2, 162 00 Prague 6, Czech Republic

[2] First Faculty of Medicine, Charles University, Salmovská 1, 120 00 Prague 2, Czech Republic

[3] Institute of Physiology, Czech Academy of Sciences, Vídeňská 1083, 142 20 Prague 4, Czech Republic

* shapoval@imc.cas.cz, horak@imc.cas.cz – corresponding authors

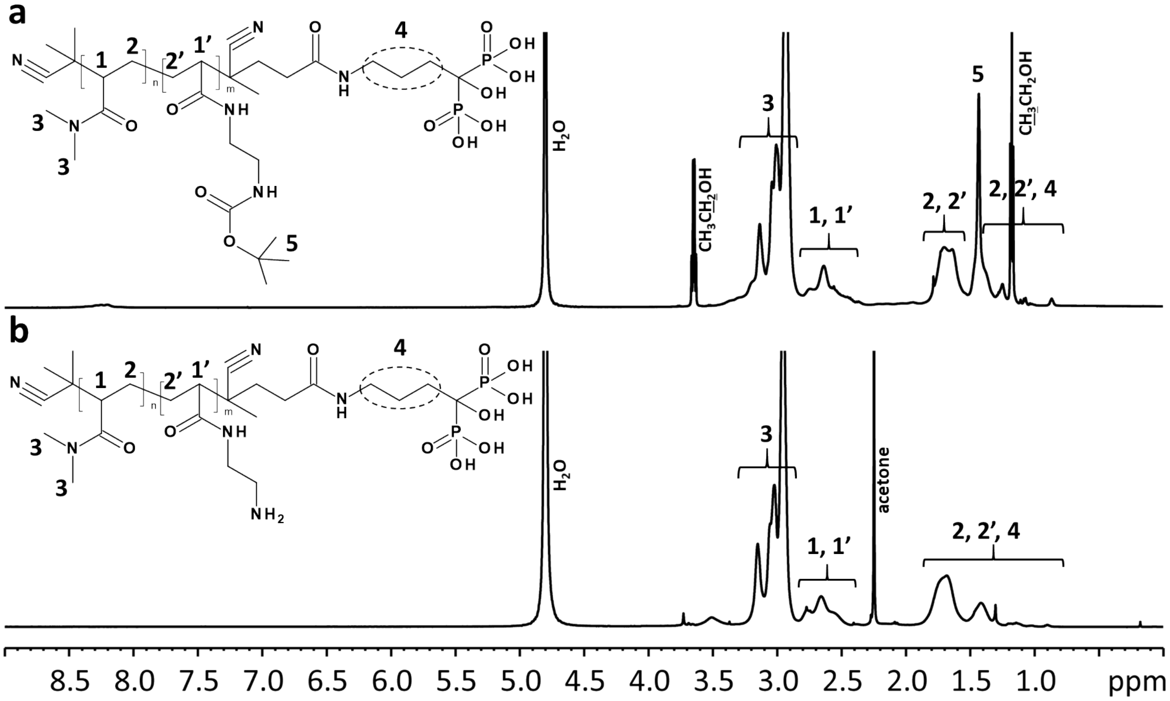

Figure S1. ^{1}H NMR spectra of (a) P(DMA-AEC-Boc)-Ale and (b) P(DMA-AEM)-Ale copolymers.

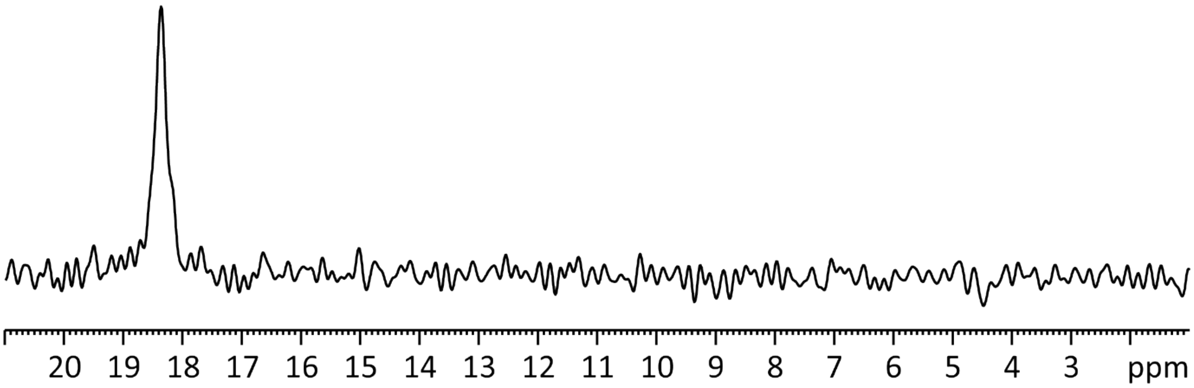

Figure S2. ^{31}P NMR spectrum of P(DMA-AEM)-Ale copolymer.

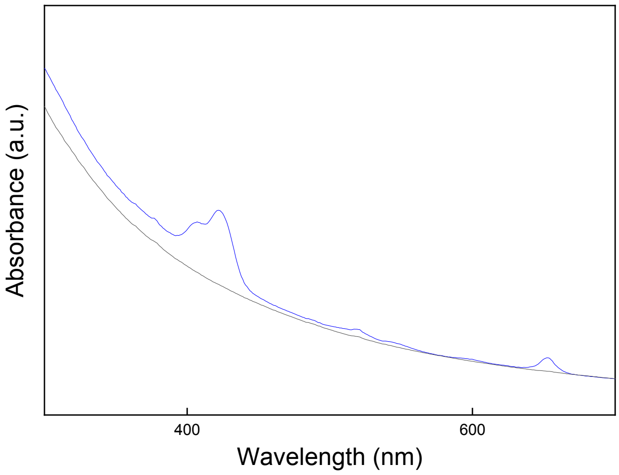

Figure S3. UV-Vis absorption spectra of UCNP@Ale-P(DMA-*co*-AEM)-PEG (black) and UCNP@Ale-P(DMA-*co*-AEM)-PEG-mTHPC particles (blue) in water (1 mg/ml).

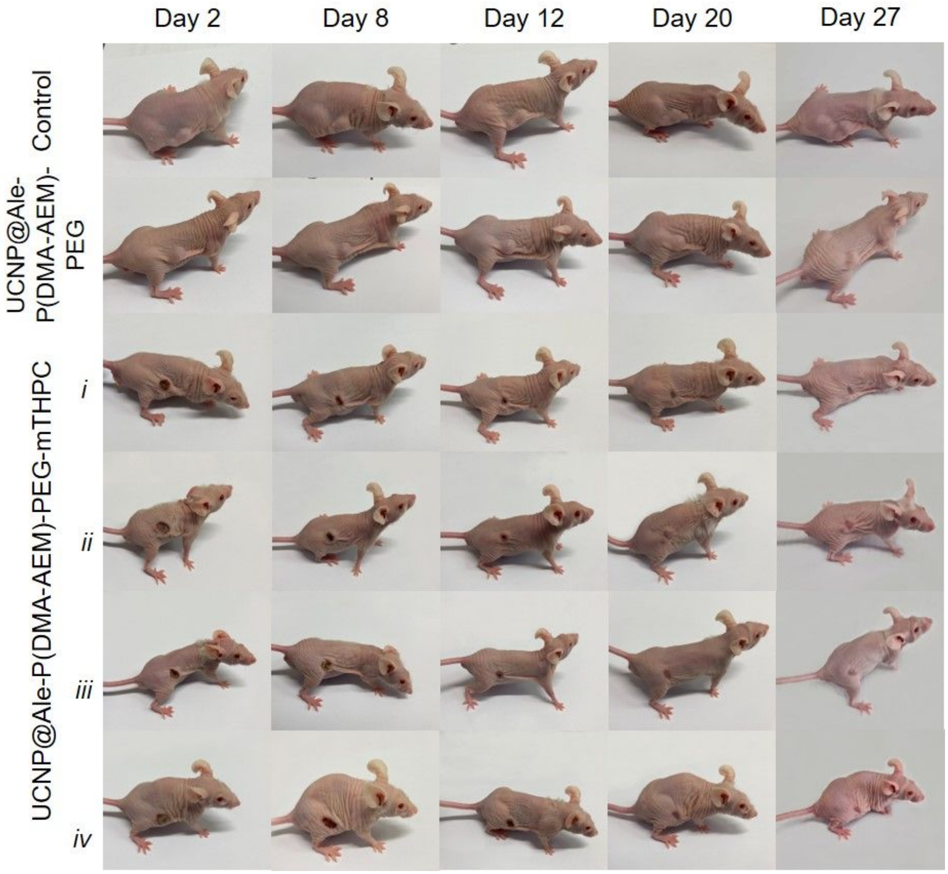

Figure S4. Nu/nu mice and a group of four nu/nu mice (*i*-*iv*) with growing human Capan-2 pancreatic adenocarcinoma treated with UCNP@Ale-P(DMA-*co*-AEM)-PEG and UCNP@Ale-P(DMA-*co*-AEM)-PEG-mTHPC colloid for different times after 980-nm NIR-induced PDT. Control - untreated mice with an intratumoral injection of PBS without irradiation.
